# Is Graphene Shortening the Path toward Spinal Cord Regeneration?

**DOI:** 10.1021/acsnano.2c04756

**Published:** 2022-08-24

**Authors:** André F. Girão, María Concepcion Serrano, António Completo, Paula A. A. P. Marques

**Affiliations:** † Centre for Mechanical Technology and Automation (TEMA), Department of Mechanical Engineering, University of Aveiro (UA), Aveiro, 3810-193, Portugal; ‡ Instituto de Ciencia de Materiales de Madrid (ICMM), Consejo Superior de Investigaciones Científicas (CSIC), Calle Sor Juana Inés de la Cruz 3, Madrid, 28049, Spain

**Keywords:** biomaterials, graphene, electrodes, glial reaction, nanocarriers, neural cells, neural stimulation, scaffolds, spinal cord injury, tissue engineering

## Abstract

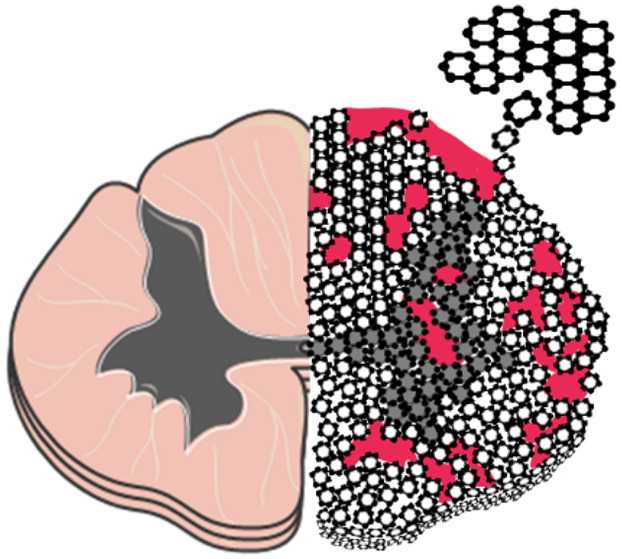

Along with the development of the next generation of biomedical platforms, the inclusion of graphene-based materials (GBMs) into therapeutics for spinal cord injury (SCI) has potential to nourish topmost neuroprotective and neuroregenerative strategies for enhancing neural structural and physiological recovery. In the context of SCI, contemplated as one of the most convoluted challenges of modern medicine, this review first provides an overview of its characteristics and pathophysiological features. Then, the most relevant ongoing clinical trials targeting SCI, including pharmaceutical, robotics/neuromodulation, and scaffolding approaches, are introduced and discussed in sequence with the most important insights brought by GBMs into each particular topic. The current role of these nanomaterials on restoring the spinal cord microenvironment after injury is critically contextualized, while proposing future concepts and desirable outputs for graphene-based technologies aiming to reach clinical significance for SCI.

Since its isolation in 2004,^1^ graphene has decisively contributed to narrow the gap between biology and electronics, boosting the development of wide-range biomedical strategies proficient to synergistically combine diagnostic, healthcare monitoring, and therapeutic modalities.^2^ The early attractiveness to integrate graphene into neural tissue engineering (TE) applications continues to date because of its inherent characteristics. In brief, graphene is a monolayer of sp^2^ hybridized carbon atoms arranged in a two-dimensional (2D) honeycomb lattice, which provides a singular electronic band able to combine metallic and semiconducting features toward a transparent zero-gap semiconductor behavior.^3^ Furthermore, it presents a high specific surface area, lightness, flexibility, and excellent mechanical integrity due to the covalent bonds between neighboring carbons. This set of properties has placed graphene as a prime candidate to match key requirements of interfaces aiming to record, manipulate and/or regenerate soft neural tissues including the spinal cord.

As a matter of fact, pristine graphene, together with other graphene-based materials (GBMs) such as graphene oxide (GO) and reduced graphene oxide (rGO), is being included in potential ground-breaking therapies targeting spinal cord injury (SCI), which continues to be one of the most disruptive medical conditions.^4−6^ From a clinical perspective, such approaches can be grouped in neuroprotective and neuroregenerative.^7^ Neuroprotective strategies aim to counteract cell death and limit injury extension, for instance, by moderating the endogenous inflammatory responses immediately triggered after trauma. Alternatively, neuroregenerative ones focus on recovering the lost functionality of the spinal cord by rebuilding its damaged neuronal circuits. Although the ideal SCI treatment should combine protection and regeneration, current standard medical procedures deeply rely on the capability of decompressive surgeries, anti-inflammatory steroids, and rehabilitation protocols to preserve residual neuronal networks in the injury site, resulting in insufficient patient recovery.^8^ Within this context, GBMs are currently boosting multifaceted strategies with the potential to overcome such inefficient therapeutics. Promising results in the field of TE reported to date can be related to (1) the capability of GBMs to promote neural stem cells (NSCs) survival and differentiation, as well as to facilitate appropriate axonal sprouting and long-tract outgrowth; (2) the ability of 3D graphene-based scaffolds to reshape the hierarchical architecture of the spinal cord and promote an effectual bridging of the injury site; and (3) the efficiency of GBMs, particularly hydrophilic GO, on counterbalancing the inhibitory SCI microenvironment with an accurate delivery of biomolecules.

More precisely, the application of GBMs in SCI therapeutics could range from the efficient delivery of pharmacological agents through the blood–spinal barrier (BSB),^9^ to the fabrication of advanced biosensing platforms capable of detecting specific SCI biomarkers to enable a more efficient monitorization of patients,^10^ to cite a few. Among all of these attractive advances, the focus given to the use of GBMs for SCI scaffolding approaches is predominant. Such prioritization is mainly related to the multitude of possible scaffolding conceptualizations provided by GBMs, considering not only the easy modulation of their physicochemical properties but also the effectiveness in shaping their dimensionality from 2D structures to 3D complex architectures suitable for conducting stimuli *in vivo*. For instance, by simply adjusting the reduction degree of rGO substrates, which can be made via chemical or thermal methodologies, it is possible to manipulate the topological defects and residual oxygen functionalities responsible for tuning the features (e.g., electrical conductivity, hydrophilicity, mechanical compliance, surface chemistry) that will directly influence neural cell responses.^11^ Moreover, the behavior of NSCs *in vitro* also proved to be highly responsive to the architecture of graphene-based scaffolds. Improved adhesion, neuronal differentiation, proliferation, and migration were found in 3D foams comparatively to 2D films, despite similar fabrication by chemical vapor deposition (CVD) and surface chemistry of both types of substrates.^12,13^ Importantly, these findings highlight the potential of 3D conductive graphene-based porous microenvironments to provide an enhanced specific surface area to optimize cellular guidance, as well as a stronger charge injection ability for electrical stimulation. It is important to note that this class of nanomaterials is able to either improve the biochemical, electrical, and/or mechanical performance of standard biomaterials (mostly natural and synthetic polymers) or trigger enhanced survival of cells in the spinal niche, unlocking neurogenesis routes and supporting, or even boosting, neuronal connectivity and functionality. They serve also to boost more efficient cell behaviors in response to certain stimuli (e.g., chemical gradients and electrical stimulation).^14,15^

Considering the emergent impact of GBMs on the development of promising therapies to target nervous system disorders, pertinent reviews have recently highlighted key strategies to build highly functional graphene-based substrates,^16,17^ fundamental interactions between GBMs and neural cells,^18^ and toxicity reports.^19^ Regarding SCI, Dominguez-Bajo *et al.*(20) specifically collected major *in vivo* findings concerning the effects of GBMs interfacing the injured spinal cord. Nevertheless, a comprehensive and multidisciplinary overview to contextualize the current position and prospective scope of these nanomaterials as therapeutic agents for SCI repair was missing. Hence, we intend to provide such contextualization. From the clinical basics of SCI and its pathophysiological features, we describe relevant neuroprotective and neuroregenerative practices targeting SCI, including standard medical procedures and ongoing clinical trials. Next, we introduce and discuss specific contributions that GBMs are making to enhance central nervous system (CNS) therapies, particularly those targeting SCI, including (1) the modulation of neural cells; (2) the delivery of therapeutic biomolecules; (3) the upcoming impact on neuromodulation and robotics, and (4) the highly promising scaffolding-based neuroregenerative approaches. Within this scenario, we highlight the singularities that GBMs offer for forthcoming spinal cord therapeutics, pointing out current clinical challenges and perspectives for future approaches. Readers are referred elsewhere for excellent reviews on pathophysiological and/or clinical aspects of SCI,^8,21^ biological and technological strategies for SCI recovery,^22^ biomaterials for SCI therapeutics,^23^ and nanotherapeutic modalities.^24^

## The Spinal Cord

**Figure 1:**
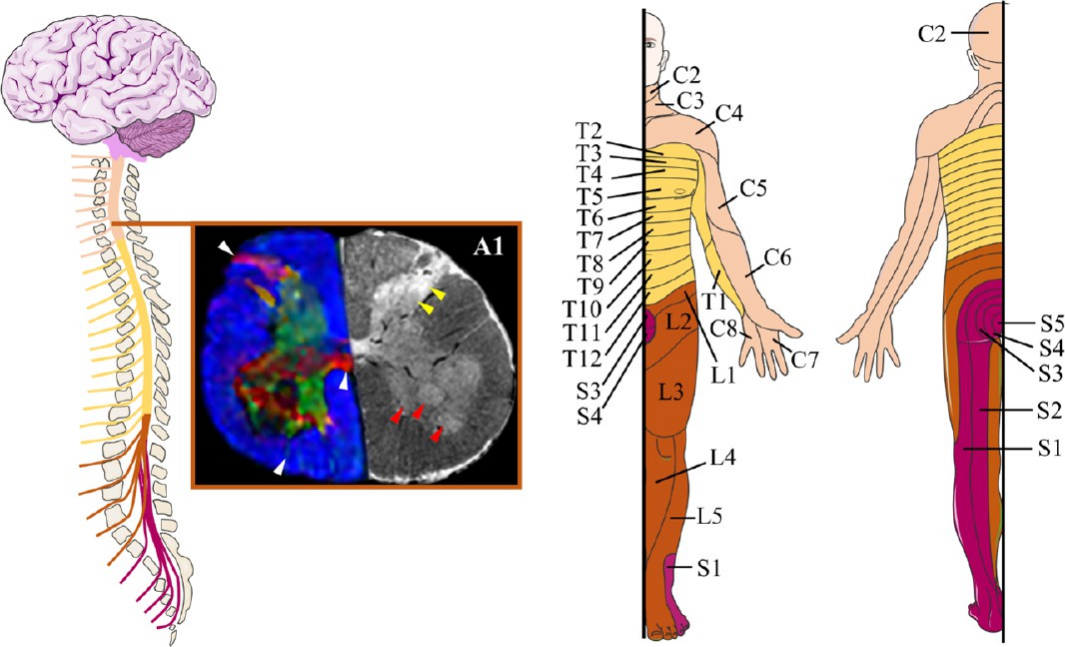
Spinal cord. Schematic overview of the spinal cord anatomy (left) and the areas of the body innervated by each spinal cord region (right): cervical (C, light orange), thoracic (T, yellow), lumbar (L, dark orange), and sacral (S, fuchsia). The figure was partly generated using and adapting Servier Medical Art templates “Neurology”, provided by Servier (https://smart.servier.com), under a Creative Commons Attribution 3.0 Unported License (https://creativecommons.org/licenses/by/3.0/). The section A1 highlights an axial MRI image of the intact human spinal cord at the fourth lumbar vertebral level. The directionally colored fractional anisotropy (left) reveals small axonal pathways including the anterior gray commissure and dorsal/ventral nerve rootlets (white arrowheads). The T2*-weighted gradient echo image demonstrates both ventral (motor) nuclei (red arrowheads), as well as dorsal (sensory) nuclei such as the nucleus proprius/substantia gelatinosa (yellow arrowheads). Adapted with permission from ref (27). Copyright 2018 Elsevier.

The spinal cord is a tubular neural structure located within the vertebral column, being continuous with the brain through the medulla oblongata. Anatomically, the spinal cord can be subdivided into cervical, thoracic, lumbar, and sacral regions, where each segment is associated with a pair of spinal nerves that enter and exit the vertebral column through intervertebral and sacral foramina (8 cervical, 12 thoracic, 5 lumbar, 5 sacral, and 1 coccygeal pairs of nerves) (Figure 1).^25^ There are substantial variations in the diameter of the spinal cord during the course of its length due to the accommodation of a larger amount of neural components responsible for the innervation of the limbs (i.e., enlargements).^26^

The organization of the spinal cord into a core of gray matter surrounded by white matter can be easily revealed from transverse sections (Figure 1, section A1).^27,28^ The gray matter, populated by both neuronal and glial cells, presents a characteristic butterfly-like shape that allows a subdivision into posterior (dorsal), anterior (ventral), and lateral horns. The posterior horns accommodate interneurons and the axons of incoming sensory neurons, while the anterior horns contain cell bodies and neurites of motor neurons. The lateral horns are considerably smaller, being the location of the somas of autonomic motor neurons. On its turn, the white matter is situated in the periphery of the gray matter, and it is primarily composed by groups of longitudinal ascending and descending myelinated axons that form sensory and motor tracts, respectively. Depending on their location, the tracts are grouped into regions called posterior, anterior, or lateral columns. The posterior columns lie between the two posterior horns of the gray matter, and they are composed of ascending axons that transport somatic sensory information (e.g., touch and pressure) from mechanoreceptors toward upper segments of the spinal cord and the brain. Differently, the anterior and lateral columns include both sensory and motor tracts capable of delivering temperature and pain stimuli to the brain and, complementarily, transporting information related to complex activities (e.g., postural control and locomotion) from the cerebral cortex to motor neurons and interneurons located in the gray matter.

As part of the CNS, the spinal cord acts as the major communication channel between the encephalon and the peripheral nervous system (PNS), through which it receives sensory inputs from the skin, joints, glands, organs, and muscles. This incoming information is then conducted to different regions in the brain, where it is processed and integrated before an action command is generated and delivered back to the spinal cord with the purpose of, by the paths defined by the PNS, prompt specific responses from limbs and/or organs in the body (e.g., voluntary movements, gland secretions).^29^ Complementarily, the spinal cord is also able to produce reflexes, called spinal reflexes, toward intrinsic neural mechanisms capable of adapting movement patterns and preventing dangerous situations. Additionally, the spinal cord contains an important spinal network referred as the central pattern generator for locomotion, which is responsible for producing and controlling various rhythmic activities such as walking, chewing, and swimming without interference from either the descending pathways or the sensory inputs.^30^ Taking these functional roles into account and considering the limited regenerative capability of the CNS, any disruption of the spinal cord neural networks could severely affect the overall function of the nervous system and, ultimately, culminate in lifetime devastating consequences for SCI patients.

## The Spinal Cord after Injury

### Classification and Epidemiology of SCI

SCI can be traumatic (TSCI) or nontraumatic (NTSCI), the cause being either an external physical impact, such as a motor vehicle accident, or chronic diseases like cancer, respectively. Additionally, SCI can be classified according to the International Standards for Neurological Classification of Spinal Cord Injury, which embraces three complementary neurological summary scores developed by the American Spinal Injury Association (ASIA) to evaluate and categorize the extension of the injury. These assessments identify the lowest caudal level where the sensory and motor components are undamaged (neurological level) and, furthermore, classify the SCI in either complete, if there is a total absence of sensory and motor functions underneath the neurological level, or incomplete, if there are sensory and/or motor functionalities that remain partially preserved.^8,31,32^ For example, the ASIA impairment scale (AIS) categorizes as AIS A the most severe case of SCI, meaning a complete loss of sensorimotor functions below the neurological level including the sacral segments. Contrarily, in incomplete lesions, patients conserve sacral sparing and are classified from AIS B to AIS E, according to their sensory and motor deficits. Such impairments often result in one of the following patterns of injury: Anterior cord syndrome, which affects the anterior two-thirds of the spinal cord and leads to a loss of sensory (pain and temperature sensations) and motor functions; Brown-Séquard syndrome, which occurs after a traumatic hemisection of the spinal cord and affects both ipsilateral (fine touch, proprioception, motor function) and contralateral (pain and temperature) sides of the injury; Central cord syndrome, which instigates a disproportionate motor deficit of the upper limbs relatively to the lower limbs due to a lesion of the central part of the spinal cord; and Posterior cord syndrome, which contrasts with the anterior cord syndrome by affecting the posterior columns of the spinal cord and, consequently, inducing the loss of both proprioception and vibration sense.

Moreover, SCI patients are generally diagnosed with either tetraplegia (preferred terminology relatively to “quadriplegia”) or paraplegia. Briefly, tetraplegia refers to a lesion in the cervical region of the spinal cord, resulting in a complete or incomplete loss of sensorimotor functions in arms, legs, and pelvic organs. Alternatively, the term paraplegia involves an injury in one of the other segments of the spinal cord (thoracic, lumbar, or sacral), which leads to a partial or total impairment of the sensory and motor functionalities below the neurological level.^33^

Relatively to epidemiology, a recent global survey estimated a total of 27 million patients living with SCI worldwide, indicating no significant variation in the age-standardized prevalence of the disease between the years 1990 and 2016.^34^ This Global Burden of Disease Injuries and Risk Factors study pointed out a global age-standardized incidence rate of 13 per 100 000 population in the year 2016. Regions with higher sociodemographic indexes presented more confirmed SCI cases (e.g., 25–26 per 100 000 population in the high-income North America, Western Europe, and Asia Pacific). However, at a country level, the most prominent incidence rates were detected in conflict/terrorism affected territories such as Syria (136 per 100 000 population). A detailed epidemiologic status of SCI is available in Chapter S1, analyzing the profiles of SCI patients together with the major etiologies of both TSCI and NTSCI across the globe. Importantly, independently of its traumatic or nontraumatic origin, the management of each SCI patient requires a continued evaluation of the injury progression and prognosis via medical imaging approaches (e.g., X-ray, computed tomography (CT) scan, and MRI) and electrophysiological studies. In the United Kingdom, the average of hospitalization and lifetime costs (e.g., rehabilitation, residential, education) is £1.12 million (approximately 1.23 million €) per SCI case, with variations associated with the age and neurological condition of the patient.^35^ Additionally, in Great Britain, after TSCI, the life-expectancy oscillates between 18% and 88% relatively to the general population, showing a particularly high mortality risk for patients during 5 years postinjury.^36^ Similar studies on Europe and United States of America (USA) populations also concluded that TSCI induces an enormous risk of early mortality, suggesting that substantial investments in healthcare and prevention programs could extend and improve the lives of patients worldwide.^37,38^

It is worth noting that this review will focus on TSCI rather than NTSCI considering three key aspects that are relevant from both medical and research perspectives: (1) the incidence of TSCI is predominant globally; (2) the pathophysiology after trauma is more foreseeable and uniform comparatively to lesions originated from degenerative disorders and cancer; and (3) TSCIs are better replicated on animal models. Therefore, in what follows, descriptive content and discussions will center on TSCI.

### Pathophysiology of TSCI

Because of its unpredictable nature, TSCI is a heterogeneous condition able to trigger dissimilar cascades of critical pathological events, depending on the type and location of the injury as well as its severity.^21,39^ Generally, any violent impact on the spine able to compress, distract, or displace the vertebrae has the potential to severely compromise the integrity of the spinal cord cytoarchitecture, leading to disruptions that can be further classified as compression injury, which occurs when a persistent compression affects the spinal cord; contusion injury, which involves a transient compressive impact that partially damages the spinal tissue components (e.g., hemorrhages, expanding necrosis and cavitation), without breaching or disrupting the surface anatomy, and laceration/transection injury, which is a cut impairment in the spinal cord provoked directly or indirectly (e.g., bone/disc fragments) by either accidents/violence involving projectiles or knifes or by an extreme spinal stretching. From a clinical point of view, contusions are the most frequent type of TSCI, the total transections of the spinal cord being extremely rare in humans but recurrently used as animal models. The degree of complexity is also associated with the extension of the lesion because TSCI can affect either a small area of tissue or, alternatively, entire segments of the spinal cord. It is also possible to diagnose multiple injuries in one patient, if the wounds were induced by gun shots, for example.

**Figure 2:**
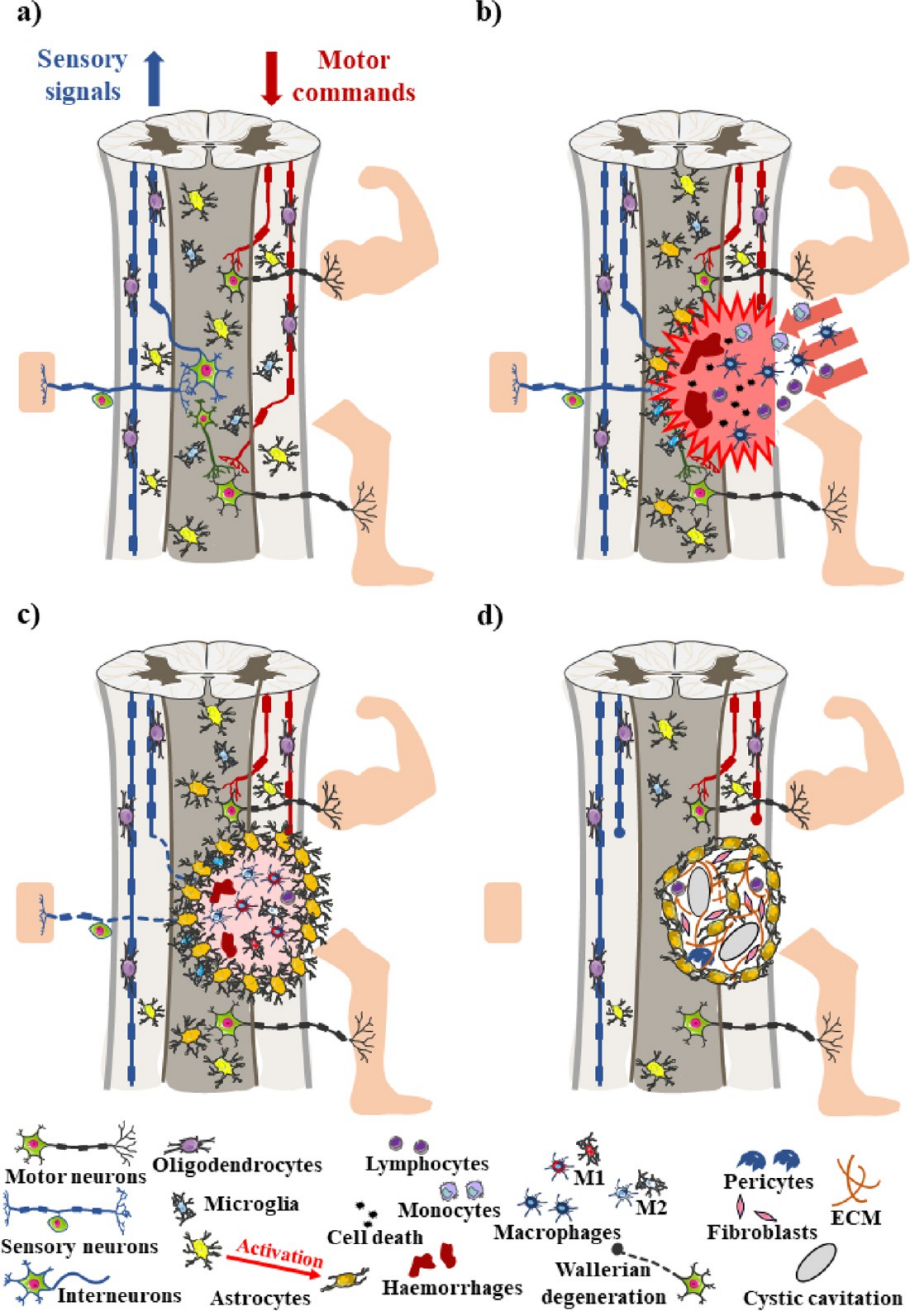
Pathophysiology of TSCI. (a) Spinal cord microenvironment before injury, including both sensory (left) and motor (right) neuronal pathways. Ascending tracks transport somatic sensory information including touch, pressure, temperature, and pain from receptors located in the skin, muscles, and internal organs through sensory neurons to the brain and upper segments of the spinal cord. Inversely, descending tracks conduct information from the cerebral cortex toward motor neurons to target peripheral effectors responsible for complex activities (e.g., movement of the upper and lower limbs). (b) Acute phase of TSCI. Trauma immediately provokes cell death and disruption of neuronal pathways, inducing the infiltration of inflammatory cells, hemorrhages, ischemia, and edema. (c) Subacute phase of TSCI. The inflammatory cascade aggravates cell death and demyelination, as M1 and M2 polarized cells trigger endogenous pro-inflammatory and pro-regenerative responses, respectively. (d) Intermediate and chronic phases of TSCI. Astrogliosis boosts the formation of the spinal injury scar, which plays simultaneously a protective and inhibitory role. At the later stages, the lesion is characterized by cystic cavitation and Wallerian degeneration. The figure was generated using and adapting the Servier Medical Art templates “Neurology”, provided by Servier (https://smart.servier.com), under a Creative Commons Attribution 3.0 Unported License (https://creativecommons.org/licenses/by/3.0/).

TSCI is usually divided into primary and secondary injuries.^8,40^ Briefly, the primary injury directly follows the traumatic event, provoking irreversible damages to the spinal cord microenvironment such as necrotic cell death (neurons and glia), abundant blood vessel damage (i.e., hemorrhages), and abrupt disruption of the ascending and descending neural pathways. Events occurring in the zone affected by the physical insult immediately initiate a secondary phase of damage able to spread to neighboring areas. Indeed, during the secondary injury, there is a torrent of biochemical events capable of boosting and propagating tissue damage, while constraining a sustainable recovery. Secondary injuries might prolong the loss of both structure and function of the spinal cord, temporally being characterized into acute (<48 h), subacute (from 48 h to 14 days), intermediate (from 14 days to 6 months), and chronic (>6 months) phases (Figure 2).

Right after TSCI, both necrosis and vascular damage initiate a complex array of interconnected and overlapped pathological mechanisms that culminate in neurological deficits. Particularly, during the acute phase (Figure 2b), the loss of intra-/extracellular ionic homeostasis promotes a massive calcium influx into cells, leading to mitochondrial dysfunction and, consequently, to critical metabolic perturbations that cause a massive production of free radicals (e.g., reactive oxygen species) responsible for triggering apoptotic signaling pathways in both neurons and glia.^41^ The ionic dysregulation is further aggravated by the toxic accumulation of glutamate around the injured site. Indeed, as this excitatory neurotransmitter is cyclically released (i.e., first wave during 20–30 min postinjury and second wave 2–3 days after injury) by dying neurons and astrocytes, its capability to provoke excitotoxic cell death also propagates during the secondary injury.^42^

Moreover, tissue destruction augments because of the unregulated passage of inflammatory cells and molecules across the compromised BSB, which is a protective and regulatory interface composed by endothelial cells, pericytes and astrocytes, that are responsible for restraining the contact between the spinal cord tissue and the blood.^43^ Such endothelial breaches accelerate the propagation of the neurological dysfunction from the injury site to adjacent areas. Specifically, hemorrhages spread in the highly vascularized gray matter, blood vessels disruption induces tissue ischemia, and the uncontrolled entrance of fluid containing cells and proteins provoke edema.^40^

The continuous cell death driven by excitotoxicity, inflammation, and ischemia triggers an aggressive anti-inflammatory response throughout the subacute phase. It results in conflicting neuroprotective and neurodegenerative effects induced by the concerted action of resident astrocytes and microglia, invading macrophages, lymphocytes, and neutrophils (Figure 2c).^44^ More precisely, the evolution of neuroinflammation is deeply related to time-dependent variations of the stimuli able to influence the polarization of macrophages and microglia into either pro-inflammatory (M1-like) or anti-inflammatory/pro-regenerative (M2-like) phenotypes. On the one hand, actions by M1 cells are earlier initiated after trauma, mainly responsible for the removal of cellular and molecular debris, including myelin, via phagocytosis. Nevertheless, as the prolonged activity of these cells instigates an abundant production of pro-inflammatory cytokines and oxidative species, collateral detrimental effects like additional cell necrosis and spontaneous demyelization are also induced. On the other hand, the later activation of cells toward the M2 phenotype contributes to the attenuation of the neurotoxic inflammatory cascade by producing cytokines and chemokines proficient to mitigate the immune response. The activity of these cells further boosts axonal growth and cell proliferation, as well as the migration of glia and progenitor neural cells, which end up differentiating predominantly into oligodendrocytes. It is important to notice that both M1 and M2 phenotypes coexist at the lesion area during a limited time period (approximately 1 week), the pro-regenerative phenotype being nearly absent during the latest stages of TSCI.^40,45,46^

Astrogliosis is another key event occurring throughout the subacute phase, during which the massive proliferation of astrocytes generates a glial scar that isolates the injury site.^47,48^ Specifically, the astrocytic activation occurs hours after trauma due to the abundant release of adenosine triphosphate and pro-inflammatory cytokines by damaged cells and reactive microglia/macrophages. The activated astrocytes become hypertrophied cells proficient to overexpress glial fibrillary acidic proteins (GFAPs), intermediate filaments, nestin, and vimentin. Furthermore, they instigate chemotaxis and influence the polarization of microglia/macrophages, establishing mutual regulation mechanisms with these cells. The proliferation of activated astrocytes starts 1–2 days after injury, extending for approximately 2 weeks until the lesion surroundings are fully covered. At this point, these astrocytes change to a scar-forming phenotype, capable of generating a highly dense layer of cells with entangled filaments. The maturation and stabilization of the astrocytic glial scar around the injury core mark the beginning of the intermediate phase, its antagonistic beneficial and inhibitory roles being continuously noticed even at the chronic stage of the disease (Figure 2d).

Although the physical barrier provided by astrocytes is determinant for the dynamics of the TSCI microenvironment, it is merely one component of the complex scar tissue. In this regard, Bradbury and Burnside^49^ have recently proposed the term “spinal injury scar” to include the entire set of cell populations and extracellular matrix (ECM) elements located not only in the lesion border (astrocytic glial scar) but also in the lesion center, which is mainly comprised by stromal-derived fibroblasts and inflammatory immune cells (fibrotic scar). The fibrotic core of the lesion plays a fundamental role in enhancing the integrity of the injured area, its formation being mostly due to the elevated activity of type A pericytes.^50^ The proliferative behavior of these cells might influence the severity of the scar pathology, as moderate pericyte-derived scarring is able to combine axonal regrowth with a constricted lesion volume, whereas the absence of type A pericyte progeny induce inefficient sealing of the lesion site.^51^ In this way, the organization of the spinal injury scar is crucial to maintain both fibrotic tissue and macrophages confined in a limited area, avoiding, consequently, an uncontrolled spread of inflammation and neurotoxic cytokines to healthy tissues.^52^ Additionally to this neuroprotective role, the scar tissue also facilitates the restoration of homeostasis as well as the recovery of the BSB functionality.^53^ However, it is also well-known that the maturation of the spinal injury scar tremendously limits the reestablishment of the native neuronal network connectivity. This hampering of axonal regeneration is due to the physical and chemical obstruction induced by the astrocytic glial scar. Specifically, noticeable variations in the ECM composition occur after injury including the downregulation of hyaluronan (also called hyaluronic acid (HA)) and the accentuated production of inhibitory molecules (e.g., chondroitin sulfate proteoglycans (CSPGs) and Nogo).^40,47^ Overall, the tissue within and around the spinal injury scar remains essentially dysfunctional after TSCI. As a matter of fact, even the complete ablation of the astrocytic glial scar at later stages of the disease (5 weeks) was incapable of stimulating the spontaneous regrowth of damaged axons.^54^ The functional recovery of the spinal cord is also considerably conditioned by the formation of cysts/cavities and the effects of progressive Wallerian degeneration occurring at intermediate and chronic phases.^8,49^

### Clinical Management and Rehabilitation of TSCI Patients

The spontaneous recovery of the spinal cord after trauma is profoundly limited by intrinsic and extrinsic factors to the neuron, as described above. Indeed, neither neuronal cells present a substantial cellular machinery to trigger efficient self-regenerative mechanisms, nor is the inhibitory ECM capable of providing sufficient biochemical and biomechanical gradients to stimulate efficient neural network regrowth. Nevertheless, in the clinical practice, a tenuous functional recovery is often observed in TSCI patients due to mechanisms of compensation developed by learning motor strategies independently of the neurological recovery (e.g., use of assistant devices to facilitate movement) and to neural plasticity processes related to the “unmasking” of synapses that were previously inactivated and the endogenous reorganization of spared neuronal circuits, including the compensatory collateral sprouting of axons.^22,55,56^ In general, endogenous attempts to repair the injured spinal cord are unable to revert the long and tragic SCI evolution, considering that patients often develop, together with sensory and motor losses, a wide range of local (e.g., Charcot arthropathy, spasticity) and systemic (e.g., cardiovascular and respiratory deficits, neuropathic pain) complications that permanently deteriorate their quality of life and independence. Therefore, under this scenario, the long-term clinical management of SCI patients deeply relies on the adaptation of lifetime interdisciplinary rehabilitation approaches, which often present limited/intangible success, and on the implementation of palliative care programs.

The initial treatment of TSCI starts locally where the accident took place by adapting standard medical procedures to each particular case. It is important to ensure resuscitation efforts, if needed, and conciliate the airway and circulatory support with a proper immobilization of the patient (e.g., cervical collar, rigid spine board). Then, the transportation to a specialized care unit should be fast for expediting the stabilization and monitorization of cardiac, hemodynamic, and respiratory processes in the patient. The efficiency and speed of these early medical procedures are crucial to augment the chances of reducing the negative effects triggered by the inflammatory cascade and, consequently, maximizing recovery.^8^ Taking this into consideration, the concept “Time is Spine” has been consolidated as a central pillar of the American Association of Neurological Surgeons and Congress of Neurological Surgeons guidelines to manage TSCI patients. It highlights the need to concentrate both diagnosis and intervention within an optimal time window to prevent and control the progression of the secondary injury.^57^

Prior to therapy, the application of diagnostic/prognostic imaging techniques, such as CT and MRI, is highly recommended to localize and classify the injury. Particularly, MRI imaging is currently an essential tool not only to evaluate the severity and extension of the injury via conventional MRI sequencers but also to promote the recognition and quantification of trauma-induced neurodegenerative features (e.g., axonal degeneration, degree of myelination) via advanced quantitative MRI techniques.^58^ Following diagnosis, the initial clinical procedures mostly rely on neuroprotective strategies, including decompressive surgeries and the administration of powerful anti-inflammatory agents.^59^ The role of decompressive surgery in TSCI treatment is to ensure the realignment of the vertebrae column, as well as the stabilization of the spinal cord and its decompression. Indeed, by relieving the compression of the spinal tissue, it prevents the escalation of neuronal damage, ischemia, and injury expansion, leading to an attenuation of TSCI pathological outcomes. Although a large number of studies concluded that an early decompression surgery (<24 h postinjury) provides the best long-term sensorimotor recovery, the ideal timing for this procedure remains a continuous debate since there are considerable clinical variations related to the lesion level/type and the sensitivity of the AIS grade system.^60,61^ In fact, there is an ongoing clinical trial (ClinicalTrials.gov ID: NCT02673320; Table S1) focused on determining the impact of early (within 48 h postinjury) and delayed (at 15 days postinjury) cervical spinal decompression surgeries on the postoperative functional evolution of TSCI patients.

Complementary to surgical interventions, the administration of methylprednisolone sodium succinate within 8 h after TSCI is considered a valuable option to regulate the production of inflammatory cytokines and attenuate oxidative stress. However, the use of this synthetic glucocorticoid is highly controversial, considering the delicate balance between its limited capacity to enhance functional recovery, even during the optimal time window of administration, and its propensity to induce infection-related side effects.^8,62^ The neuroprotection of TSCI patients could be further improved by applying other therapeutic techniques such as mean arterial pressure augmentation and hypothermia, leading, respectively, to the optimization of spinal cord perfusion and to the moderation of the degenerative inflammatory cascade.^63,64^ In the case of systemic hypothermia, promising results obtained in previous clinical trials^65^ support a study (ClinicalTrials.gov ID: NCT02991690) that intends both to confirm the efficacy and safety of this therapy (intravascular; 33 °C for 48 h) in the early management and treatment of cervical TSCI patients and to validate its potential for improving neurological outcomes.

**Figure 3:**
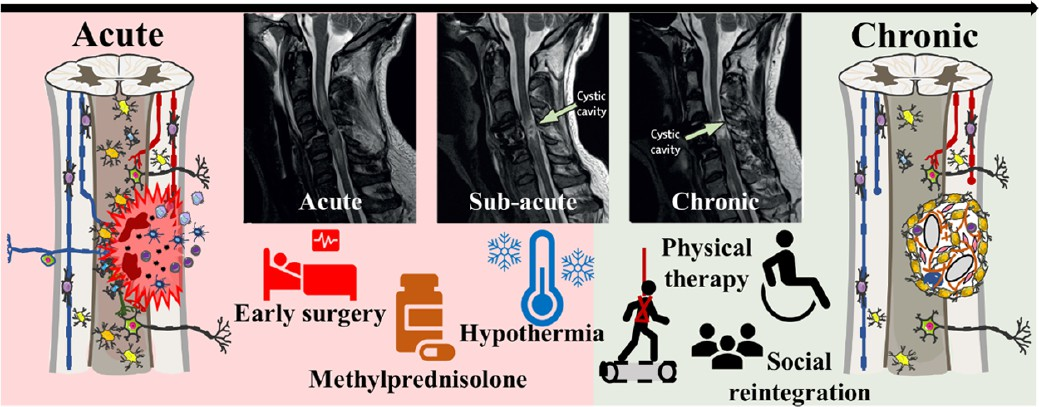
Standard clinical evolution of TSCI patients, going from diagnostic, through therapy to rehabilitation. The figure was partially generated using and adapting the Servier Medical Art templates “Neurology”, provided by Servier (https://smart.servier.com), under a Creative Commons Attribution 3.0 Unported License (https://creativecommons.org/licenses/by/3.0/). MRI data adapted with permission from ref (58). Copyright 2019 Elsevier.

Generally, the rehabilitation of TSCI patients aims to conciliate the prevention of neurodegenerative outcomes and the optimization of functional recovery via compensatory mechanisms. Such methodologies should be adapted to each stage of the disease in order to maximize: (1) the independence of the patient when facing daily routines like eating, dressing, and using a wheelchair; (2) the emotional stabilization of the patient during the acceptance of a different lifestyle (e.g., recreational and sexual activities); and (3) the reintegration of the patient in the society.^66^ Thus, TSCI rehabilitation is a long and multidisciplinary effort that requires the dedicated involvement of physicians, physiotherapists, occupational therapists, psychologists, and social workers, as well as appropriate logistics for physical therapy, including strength training, cardiovascular and respiratory exercises, mobility practicing, and muscle stretching.^8^ For example, body weight-supported locomotor training (BWSLT) is a common rehabilitation practice in which the patients are supported by machines and/or therapists while attempting locomotion in open ground or on a treadmill (e.g., Lokomat). In a recent study, it was reported that, after 120 sessions of BWSLT, patients suffering from incomplete TSCI improved their upper and lower motor strength, functional activities and autonomic functions (e.g., bowel and bladder functionalities), enabling the reduction of hospitalization time and healthcare costs.^67^ Side by side with physical therapy, it is also necessary for TSCI patients to be integrated into occupational therapy programs for augmenting their long-term functional independence. The standard stages of clinical management and rehabilitation in TSCI patients are summarized in Figure 3.

## The Promise of GBMs for Cell-Based Therapies in SCI

### Stem Cells for SCI Therapeutics

Cell transplantation proposes not only to replace the damaged tissue with functional cells but also to modulate the endogenous response following SCI, including the stimulation of dormant neuroregenerative mechanisms. Indeed, this therapeutic concept relies on the specific interactions established between the particular transplanted cell type and the host cell populations to mediate improvements such as axon regeneration, immunomodulation, neuroprotection, neural plasticity, and remyelination.^68^ For example, it has been hypothesized that the transplantation of autologous human Schwann cells could optimize neurite outgrowth while counterbalancing some harmful effects of the SCI inflammatory process like demyelization and cystic cavitation. The use of these PNS glial cells is already being validated by clinical trials focused on safety issues (ClinicalTrials.gov IDs: NCT01739023 and NCT02354625; Table S1), with preliminary results indicating that, after transplant, patients did not experience postsurgical complications, additional tissue damage, or serious impediments to participate in home-based training programs.^69,70^

Specifically, the transplantation of stem cells into the injured area, followed by a controlled differentiation process into both neurons and glial cells, could provide an interesting set of repair mechanisms to counterbalance SCI damage. However, there is a specific assemblage of clinical (e.g., immune response, tumorigenicity) and ethical challenges associated with each stem cell population, besides the difficulty to fully understand the biological mechanisms responsible for triggering functional improvements. So, a large number of ongoing investigations intend to institute reliable benefit/risk balances for numerous candidates for transplantation, ensuring the selection of appropriate cell-based methodologies for each targeted neuroregenerative process.^71^ For instance, human embryonic stem cells (hESCs), ideal candidates for neuroregeneration due to their ability to differentiate into specialized CNS cells, present serious ethical issues related to the use of human embryos.^72,73^ To avoid these ethical constraints, autologous cell therapies based on induced pluripotent stem cells (iPSCs) are being received with huge interest regardless of some associated drawbacks related to their limited reprogramming efficiency and the risk of mutagenesis.^74^ A study conducted by Lu *et al.*(75) reported that implanted iPSC-derived NSCs were proficient to successfully colonize the lesion site and, from there, promote a long-distance axonal outgrowth capable of forming interconnected neural networks with rodent host axons. Importantly, there is a human clinical trial involving the use of iPSCs for spinal cord therapy already approved by Japan authorities (UMIN ID: UMIN000035074).^76^ In this study, which will be completed in 2023, the iPSCs are transplanted 2–4 weeks after SCI into AIS A patients with the purpose of evaluating the safety and efficiency of the methodology.

The multipotent state of mesenchymal stem cells (MSCs) and NSCs motivates a potential dual role, assuming neuroprotective purposes and/or neuroregenerative functions supported by the astrocytes, neurons, and oligodendrocytes generated after differentiation. As the available sources of autologous MSCs (e.g., adipose tissue, amniotic liquid, bone marrow, umbilical cord) imply minor ethical concerns, they are being highly explored to release paracrine biomolecules (e.g., anti-inflammatory cytokines, growth factors) and to actively rebuild the spinal cord *in vivo* after neuronal differentiation.^77,78^ Alternatively, the transplantation of NSCs, which are precursor or stem cells isolated from the CNS, has evidenced encouraging neuroregenerative mechanisms including neuronal differentiation, axonal outgrowth, and formation of functional synapses in SCI animal models.^79,80^ For example, Rosenzweig *et al.*(81) have recently used a primate SCI model to validate the capability of human spinal cord-derived NSCs to repair the injured spinal cord cellular microenvironment. The results showed that NSCs differentiation into both neurons and glia assisted the successful integration of the graft into the host tissue, leading to the generation of mature (over 9 months) and interconnected neuronal networks responsible for augmenting forelimbs functionality. The safety and efficacy of both MSCs and NSCs as SCI therapeutic agents are currently being ascertained in clinical trials (ClinicalTrials.gov IDs: NCT01676441 and NCT01772810, respectively).

In addition to the ethical and clinical challenges related to the usage of stem cells, particularly hESCs, other questions still need to be addressed before cell-based treatments for SCI can be indisputably validated: (1) to fully understand the specific biological mechanisms responsible for boosting particular differentiation patterns, regulating cell–cell interactions and stimulating neuroregeneration in the injured microenvironment; (2) to standardize and regulate good-practicing manufacturing rules regarding the collection, engineering, and transplantation of each cell type with the purpose of guaranteeing the efficacy, quality, reproducibility, and safety of the therapeutic methodologies; (3) to define the optimal therapeutic time-widow for cell transplantation; and (4) to elucidate the benefits of combining cell-based treatments with other therapeutic strategies to improve the recovery of SCI patients.

### GBMs Interfacing Neural Cells

It is still early to make a conclusion about the efficacy of interfacing GBMs with intracellular organelles to optimize the output of cell-therapies targeting SCI. Any progress in this matter must rely on substantial comprehension of the positive and negative effects induced by these nanomaterials into the cellular machinery, as well as robust anticipation of potential short- and long-term toxic responses *in vivo*. It is important to notice that comparisons among studies are difficult due to the enormous variability of protocols and parameters explored, particularly the chosen cell lineage, GBMs concentration, time points, and fabrication methodology. Indeed, there are conflicting results relatively to the nanotoxicological profiles of GBMs, those mainly being associated with the dissimilar physicochemical properties of the tested nanomaterials (e.g., lateral dimensions, number of layers, oxidation state, and shape).^17,82−84^ Such features are responsible for inducing divergent interactions with the cell membrane and, therefore, instigating different intracellular mechanisms that could provoke negative outcomes like inflammation, oxidative stress, genotoxicity, and cell death. Based on this, research efforts to respond to the challenges that arise by SCI mainly concentrate on elucidating the distinct mechanisms underlying the responses of stem cells and CNS neural cell populations when interfacing GBM nanosheets. To discriminate the major particularities of the studies reviewed here, please refer to Table S2.

### Specific Interactions with Neural Stem Cells

Theoretically, the controlled delivery of stem cells in the injured area could lead to a sustainable neuronal differentiation *in situ* and, subsequently, to the establishment of functional multisynaptic connections suitable for bypassing the lesion, notwithstanding its heterogeneity and inhibitory pathophysiological profile.^85^ Such an aim presupposes accurate control not only over the “stemness” and differentiation pathways of the delivered stem cells but also over their migration, survival, and integration in the host tissue. Similarly to other organic and inorganic nanoparticles, GBMs are gradually being introduced as promising therapeutic agents either for regulating stem cell fate or for real-time image-guiding cell tracking *in vivo*.^86^

The hydrophilic character of oxygenated GO, together with its inherent high levels of biocompatibility and tunability, tendentially favors enhanced interactions with the cell membrane, driving an easier internalization. For example, GO sheets significantly boosted the differentiation of mouse ESCs (mESCs) into dopamine neurons via a stromal cell-derived inducing activity protocol, in contrast to the negligible effects triggered by other carbon-based nanomaterials such as rGO and multiwalled carbon nanotubes (MWCNTs).^87^ In this study, although the authors described GO dosage-dependent differentiation, the molecular pathway responsible for augmenting the neuron-related gene expression was not detailed. Contrariwise, Jing *et al.*(88) reported that a suspension of GO nanosheets was proficient to reduce the integrin signaling of mESCs, resulting in the maintenance of their self-renewal capacity. More precisely, as it was indicated by the downregulation of vinculin and the subsequent decline in the expression of mitogen-activated protein kinase (MEK1), GO significantly influenced the activity of the focal adhesion proteins responsible for mediating differentiation. This outcome was recently correlated to the number of layers of GO nanosheets as well as to their preferential distribution at the outside of the cell membrane, where the multilayered GO configuration (thickness of 3.2 nm) was able to better adsorb ECM components like fibronectin and, therefore, compromise its interaction with integrin receptors.^89^ Contrarily, it was highlighted that the lower surface energy of monolayered GO (thickness of 0.7–1.2 nm) was insufficient to substantially restrain fibronectin–integrin coupling, thus promoting a decrease of the differentiation potential of mESCs.

The preferential attachment of GO nanosheets onto the outer of the plasmatic membrane of human fetal NSCs (hfNSCs) was also pointed out by Kim *et al.*(90) as a key event to enhance cell–cell and cell–matrix interactions responsible for regulating the differentiation potential of neurospheres *in vitro*. The presence of GO boosted the expression of adhesion molecules (e.g., E-cadherin) and the secretion of ECM components (e.g., fibronectin) by hfNSCs neurospheres, leading to either the maintenance of their self-renewal capability or an accelerated differentiation process (e.g., neurons, astrocytes, and oligodendrocytes) depending on the applied culture conditions (e.g., substrate and culture medium composition). The performance of GO nanosheets could be further adapted by regulating their dimensions. For example, nanoparticles with a mean size of 663 nm improved the ability of mouse NSCs to maintain their pluripotency *in vitro*, while larger GO (4651 nm) augmented the expression of neural markers such as Tuj1 (neurons) and GFAP (astrocytes).^91^ Moreover, GO nanosheets, particularly the larger ones, were able to encourage neurosphere migration through the substrate, being also proficient to boost the extension of neurites in opposition to the smooth and spherical morphology observed in the control.

The limited amount of studies focused on interfacing stem cells directly with graphene or rGO nanosheets can be associated with the higher risk of generating unstable cell-nanomaterial interactions because of the hydrophobic profiles of these GBMs and their tendency to aggregate in aqueous solutions. Notwithstanding, Akhavan and co-workers tested the effects of rGO nanosheets on the viability of human MSCs (hMSCs), comparing their performance with GO and other graphene-based structures like single-layer rGO nanoribbons^92^ and rGO nanoplatelets.^93^ The results revealed that for an exposure period of 24 h, contrary to rGO nanoribbons and nanoplatelets, neither rGO nor GO nanosheets induced genotoxicity in hMSCs independently of the size and concentration of the nanosheets. Instead, although GO proved to be ∼30% less cytotoxic than rGO, both GBMs triggered oxidative stress mechanisms in a size- and concentration-dependent manner, the integrity of the cell membrane being slightly affected by rGO at high concentrations (100 μg/mL). Interestingly, GO nanosheets with sizes above 20 nm show high levels of cytocompatibility with stem cells.^94^

On the basis of this finding, GO appears to be a promising candidate to integrate upcoming stem cell-based regenerative routes targeting SCI due to its proven capacity to potentiate specific differentiation pathways of grafted stem cells, leading to repopulation of the injured spinal cord. Despite this progress, it is still necessary to unearth (1) safety and functional boundaries regarding concentration, dimensions, shape, and oxygen content of GO-based nanomaterials both *in vitro* and *in vivo*; (2) underlying mechanisms that follow cell uptake of GO nanosheets by stem cells and the identification of subsequent medium/long-term effects; and (3) precise methodologies and parameters suitable for regulating potency and differentiation patterns without generating collateral cytotoxicity via reactive oxygen species and/or physical damages of the cell membrane.^84,95,96^ Furthermore, the near future therapeutic reach of GBM–stem cell interactions will be determined by a comprehensive understanding regarding their response when interfacing stimuli from the SCI microenvironment, as well as their competence to maintain *in situ* the planned triggering of complementary differentiation routes toward cells with relevant functions in the spinal scenario.

Critically, from a clinical point of view, GBMs must be able to inhibit undesirable collateral effects of stem cell transplantation such as the generation of tumors due to uncontrolled cell proliferation and migration. In this regard, there is a growing interest in adapting the properties of graphene quantum dots (GQDs) to cell labeling and real-time monitoring, providing not only insights regarding the distribution of stem cells after implantation but also important feedback concerning their performance via structural and functional imaging.^97^ Shang *et al.*(98) have already confirmed the feasibility of GQDs to be successfully internalized by human NSCs via receptor-mediated endocytosis without compromising viability, proliferation, self-renewal capacity, and differentiation *in vitro*. In another study, GQDs were proven to label human adipose-derived stem cells *in vivo*, allowing their accurate tracking and quantification after 24 h postimplantation in a rat model.^99^ The possibility to adapt a similar strategy to cell-therapies targeting SCI could eventually permit following the biodistribution of one or more transplanted stem cell lines within the lesion area, as well as closely monitoring the subsequent interactions with the host tissue in real-time. Hence, it could be feasible to efficaciously evaluate regenerative outcomes and subsequently adjust the treatment according to specific requirements of the SCI progression. This hypothetical scenario is being presently endorsed by the advantages provided by GQDs over other QDs and standard fluorescent probes, including a nonzero bandgap, an exceptional photostability *in vivo*, high dispersibility, a large functional surface, and, generally, high levels of biocompatibility both *in vitro* and *in vivo*.^100,101^ The majority of the studies pointed out that, after cell internalization, GQDs localize in both the cytoplasm and the nucleus without causing noticeable levels of genotoxicity. However, the inclusion of these GBMs into stem cell therapy is still premature considering that their intracellular impact is poorly known yet. For example, it was noted by Ku *et al.*(102) that the uptake of GQDs did not disturb the viability and pluripotency of mESCs but inhibited DNA methylation of the pluripotency factor Sox2, leading to a delay of the differentiation process into embryonic bodies. In addition to safety concerns, the mechanisms behind the photoluminescence of GQDs (mainly driven by shape, size, edge state, and surface chemistry) need to be well-defined and controlled with the purpose of enhancing the quality and long-term imaging of the labeled stem cells even during deep tissue penetration.^103^ Further attention must be given to additional limitations of stem cell labeling regarding the dilution of the signal during cell division and the undeniable identification of living cells during tracking.^104^

### Specific Interactions with Neurons

**Figure 4:**
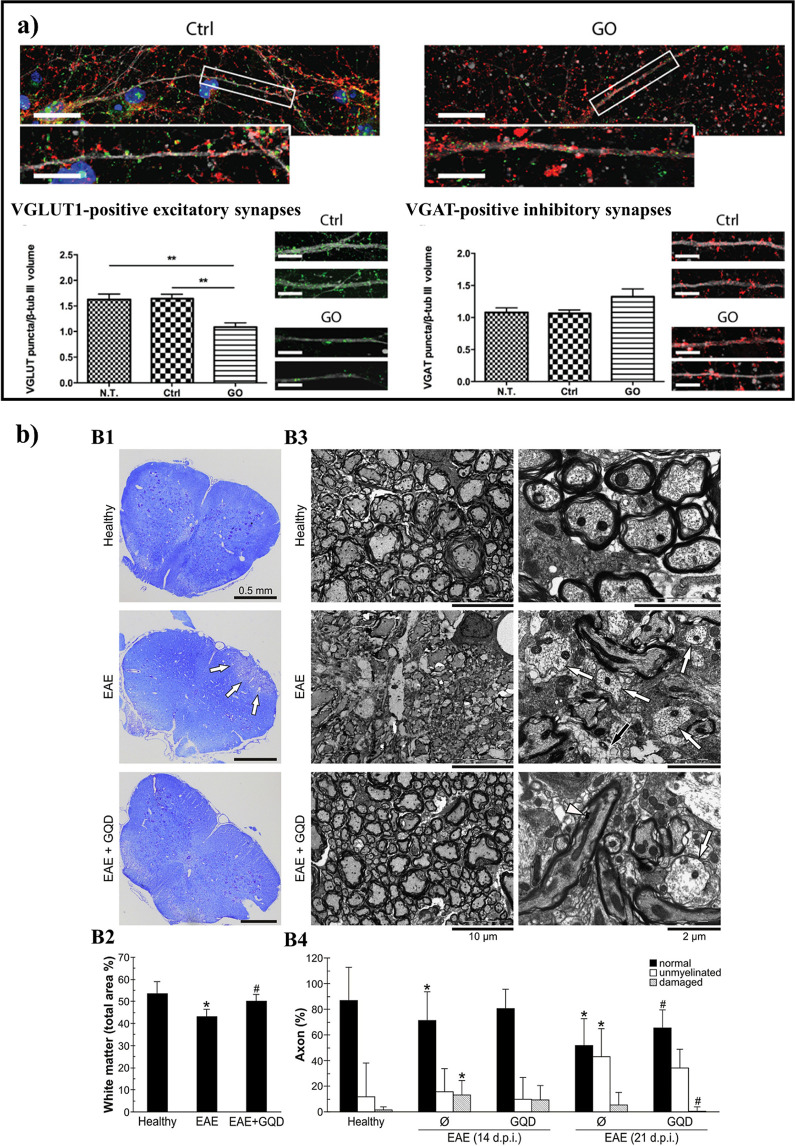
GBMs and neuronal cells. (a) Internalization of GO nanosheets by cortical neurons. Representative images of cultured neurons at 17 days *in vitro* (top) and the density of VGLUT1-positive excitatory synapses and VGAT-positive inhibitory synapses in untreated (N.T.), vehicle-treated (Ctrl), and GO-treated neurons (bottom). Neurons stained for β-III tubulin (gray), vesicular glutamate transporter (VGLUT1, green), and vesicular GABA transporter (VGAT, red). Nuclei were stained with DAPI. Scale bars = 5 μm. Adapted with permission from ref (105). Copyright 2016 American Chemical Society. (b) GQDs reduce demyelination and axonal damage in the spinal cord during autoimmune encephalomyelitis (EAE). Lumbosacral spinal cord samples were collected at day 14 (B1, B2, B4) or day 21 (B3, B4) from healthy (PBS-injected) and EAE rats treated or not with graphite-derived GQDs (10 mg/kg/day). Demyelination was assessed by Luxol fast blue staining (*n* = 5 rats per group, cumulative score) (B1, B2) or TEM (*n* = 3 rats per group; day 14 cumulative score 7.8 ± 2.9 vs 3.0 ± 2.0; day 21 cumulative score 17.0 ± 6.5 vs 5.5 ± 5.3 in control EAE vs GQDs treatment) (B3, B4). Representative micrographs are shown in (B1) and (B3; low magnification–left panel, high magnification–right panel), with white arrows, black arrow, and white arrowhead pointing at demyelination, axonal damage, and GQDs, respectively. The quantitative analysis of demyelination by Luxol fast blue staining and TEM is presented in (B2) and (B4), respectively (*p < 0.05 vs healthy control; #*p* < 0.05 vs EAE control). Adapted with permission from ref (115). Copyright 2019 Elsevier.

Nowadays, little is still known about the cytotoxic, molecular, and physiological effects triggered by exposing neurons to GBMs.^18^ For instance, Bramini *et al.*(105) recently explored the capability of both graphene and GO nanosheets to influence the response of cultured primary neurons, highlighting some important findings. First, a substantial number of nanosheets were not able to enter the cells due to aggregation. Second, the internalized nanosheets followed the endolysosomal pathway without affecting either viability or morphology of the neurons. Last, only long-term exposure to GO (2 weeks) was capable of markedly influencing neuronal activity, favoring inhibitory synapses rather than excitatory neurotransmissions (Figure 4a). In a different study, Rauti *et al.*(106) further corroborated that, contrary to cytotoxic GO big flakes (10–15 μm), GO nanosheets with a smaller size (50–500 nm) were biocompatible and proficient to interfere with the presynaptic glutamatergic terminals of neurons toward the downregulation of excitatory synaptic activity. The same group conducted an *in vivo* study to analyze the impact of intrahippocampal delivery of GO in a rat model.^107^ The authors proved that the presence of GO nanosheets into the presynaptic membrane induced an initial release of glutamate, boosting excitatory synaptic activity, followed by an obstruction of the presynaptic exocytosis process that significantly limited glutamatergic neurotransmission. Additionally, the inhibitory (GABAergic) synapses were unaffected, and the nanomaterial was nearly absent of the injected area after 72 h, avoiding any aggressive microglia activation.

Indeed, compared to other potential therapeutic agents such as charged liposomes and CNTs, GO nanosheets presented the best behavior in terms of mediating inflammation after 7 days postintracerebral injection, notwithstanding the common ability of carbon-based nanomaterials to avoid neurodegeneration and microglia activation.^108^ An intraspinal administration of GO nanosheets was able to replicate similar results, including a decrease of excitatory synapses in the spinal neural network after injection and a subsequent decline on the locomotor activity in zebrafish embryos.^109^ This promising performance of GO as a biocompatible inhibitor of the glutamatergic neurotransmission could support its delivery in neuroprotective approaches targeting SCI, particularly for attenuating the glutamate-induced excitotoxicity occurring during secondary injury. Yet, it is important to notice that the ability of GO to produce CNS toxicity is still controversial, as evidenced by an *in vivo* study in which this nanomaterial was capable of translocating from the aqueous environment to the brain of zebrafish larvae, where it subsequently provoked Parkinson’s disease-like symptoms such as disrupted locomotive activity and loss of dopaminergic neurons.^110^

Contrary to GO, the presence of rGO nanosheets in the zebrafish spinal cord triggered an increase in the locomotor performance directly associated with an augmentation of the local synaptic activity.^111^ Interestingly, a delayed but long-term impact of rGO nanosheets on the swimming performance of zebrafish embryos was evidenced (i.e., effects detected as early as 4 h of exposure and still noticeable after 24 h). Conversely, GO flakes induced earlier (after 2 h of exposure), but not persistent (absent after 24 h of incubation), inhibitory effects. The significant impact of rGO nanosheets on neural circuitries was further confirmed with rat postnatal hippocampal cultures *in vitro*. Indeed, results showed that internalized rGO nanosheets boosted the amplitude and frequency of spontaneous postsynaptic currents, as well as the number of presynaptic vesicles containing glutamate, leading to an enhancement of neuronal network excitability. This particular finding appears to be in contrast with a recent study carried out by Kang *et al.*,^112^ who described the ability of neuronal cells (PC12 cell line and embryonic cortical neurons) to oxidize rGO nanosheets via intracellular reactive oxygen species *in vitro*. The higher oxygen content of the resultant nanomaterials further disrupted actin filaments dynamics, leading to a depression of neurotransmission confirmed by the lower amplitude and frequency of the excitatory spontaneous postsynaptic currents. Noteworthy, the discrepancies between the outcomes of both studies could be majorly related to the selected cell types (postnatal hippocampal neurons vs PC12/embryonic cortical neurons), the physicochemical properties of the rGO nanosheets used (total oxygen: 6.1% vs 11.4% and size: from 300 nm to a few μm vs 98.4 ± 21.3 nm, respectively), and the treatment conditions applied (rGO concentration: 10 μg/mL vs 20 μg/mL and time of exposure: 6–8 days vs up to 12 h). Based on preliminary nanotoxicological profiles of rGO already reported, the route of administration might be also critical to ascertain biocompatibility. While rats showed no signs of CNS toxicity after intravenous injection,^113^ a short-term decline in their locomotor activity and neuromuscular coordination could be found when rGO nanosheets were orally administrated.^114^

Alongside with the modulation of neuronal cells by GBM nanosheets, the delivery of GQDs into the injured spinal cord might hold a significant reparative potential. Among the scarce reports focused on CNS diseases, Tosic *et al.*(115) used an autoimmune encephalomyelitis rat model to evaluate the performance of GQDs when counteracting neuroinflammation *in vivo*. The results included the analysis of spinal cord tissue, revealing that the internalization of GQDs into both immune and CNS cells boosted the mitogen-activated protein kinase/protein kinase B (MAPK/Akt) signaling pathway and, therefore, induced substantial neuroprotective effects on both neurons and oligodendrocytes. More precisely, the white matter of the untreated rats presented distinctive marks of inflammatory damage including demyelination, axonal degeneration, and high cell death (Figure 4b). Future studies should assess the neuroprotective reach of GQDs on preserving the spinal cord microenvironment during early SCI stages. Particularly, it is mandatory to test the capacity of GQDs to (1) prevent the inflammatory cascade that follows the rupture of the BSB at the acute phase; (2) tune M1/M2 polarization along the lesion progress; and (3) mitigate features of neural degeneration such as cell death and demyelination. In this sense, Kim *et al.*(116) proved that GQDs were proficient to penetrate through the brain–blood barrier, without inducing toxicity and ameliorate some critical Parkinson’s disease pathological outcomes such as neuronal death, low synaptic protein levels, and mitochondrial dysfunction. Further work in this topic is highly encouraged.

### Specific Interactions with Glial Cells

As glial cells are pivotal players in the regulation and support of neural circuitries functioning, the reestablishment of the complex and heterogeneous array of local cell–cell interactions becomes a milestone for the success of SCI therapies. In this scenario, GBMs could soon emerge as promising agents to adjust, or even boost, neuroglia responses at the molecular, structural, and functional level.^117^

Regarding specific features of GBM–glia interactions, Chiacchiaretta *et al.*(118) have demonstrated that the internalization of graphene and GO did not influence the viability of astrocytes *in vitro*, as both GBMs followed an endolysosomal pathway after internalization. Yet, astrocytes changed their cytoskeleton rearrangement from a standard epithelioid morphology to an asymmetric shape with long processes, acquiring a more differentiated state. Regarding their performance, in contrast to the graphene-treated cells, the astrocytes interfacing GO nanosheets were proficient to regulate the extracellular microenvironment and neuronal activity. In detail, the intracellular presence of this nanomaterial triggered the upregulation of K^+^ buffering and the uptake of glutamate, leading to a population of astrocytes suitable for accelerating the maturation of cocultured neurons and enhancing the density of inhibitory synapses. The molecular modulation triggered by GBMs in astrocytes were also covered by Bramini *et al.*,^119^ who associated the different chemical compositions of graphene and GO nanosheets to the generation of distinct proteonic and lipidomic profiles. Indeed, a more oxygenated surface of GO induced 255 alterations in proteins of intracellular routes against 80 proteins for graphene. Moreover, GO-treated astrocytes showed an upregulation of phosphatidylcholines and a downregulation of triacylglycerols, graphene being responsible for activating the opposite modulation. The role of GO in the regulation of calcium dynamics was also emphasized, considering its ability to increase cholesterol levels in the cell membrane and, consequently, to disturb the activity of Ca^2+^-binding proteins to impair the spontaneous and evoked Ca^2+^ signaling. Following this trend, a recent study highlighted that astrocytes treated with GO nanoflakes increased both the production and the release of microvesicles involved in intercellular communication *in vitro*.^120^ The posterior isolation and delivery of these GO-derived microvesicles into a cortical primary culture further triggered an enhancement of synaptic activity (increase of postsynaptic currents frequency), together with a significant softening of neuronal mechanical properties, which was directly associated with the vesicular fusion occurring in the plasma membrane.

The maintenance of viability and low reactivity levels in astrocytes after GBMs uptake is a very promising indicator for the inclusion of these nanomaterials in future SCI neuroprotective strategies. Eventually, the delivery of GO nanomaterials into the injured site during the acute/subacute phase could contribute to counterbalance the homeostatic deregulation that leads to massive cell death. To support such hypotheses, it is important not only to focus on modulating neuron-astrocyte interactions but also to understand the response of macrophages and microglia when interfacing GBMs both *in vitro* and *in vivo*. Such insights are critical to determine the range of fabrication and administration conditions suitable for boosting effectiveness while mitigating the neuroinflammatory potential of these nanomaterials, as well as for minimizing the risks associated with their biodegradation *in vivo*. Even though there is only a small number of studies addressing CNS defensive mechanisms against GBMS, a recent report pointed out that, when tested with macrophages *in vitro*, rGO nanosheets diminished the levels of reactive oxygen species and the release of proinflammatory cytokines comparatively to GO nanosheets after 24 h.^121^ Only macrophages exposed to an elevated concentration of GO (10 μg/mL) were proficient to trigger a significant expression of CD80, which is a common surface marker of pro-inflammatory phenotypes. In a more complex setup involving a 3D mouse organotypic spinal cord culture, long periods of exposure (2 weeks) and high concentrations (25 and 50 μg/mL) of GO nanosheets led to substantial microglia proliferation without provoking an accentuated release of pro-inflammatory molecules nor inducing astrogliosis.^122^ Although neuronal viability was maintained, both excitatory and inhibitory synapses were downregulated, opposing the selective response of hippocampal neurons just limiting glutamatergic neurotransmission *in vitro* and *in vivo*.^107^ Considering the similitude between both tested GO nanosheets, this particular disparity in synaptic activity could be related to either the CNS tissue investigated (spinal cord vs brain), the stage of neuronal development (postnatal vs embryonic), or to the modality adopted to deliver the nanomaterials (delivery from fibrin glue vs injection).

## The Promise of GBMs for Pharmacological Therapies in SCI

### Pharmacological Approaches for SCI Therapeutics

Recent studies have documented that some medications already approved by the Food and Drug Administration (FDA) of the USA, such as Minocycline and Riluzole, appear to be auspicious candidates for neuroprotective strategies. On the one hand, Minocycline is a structural analogue of the tetracycline antibiotic, being a proficient drug to induce anti-inflammatory, antioxidant, and antiapoptotic responses following TSCI.^123^ Indeed, both animal models and clinical trials (ClinicalTrials.gov ID: NCT00559494, Table S1) have already proved that, by attenuating microglial activation, the administration of Minocycline contributes to decrease the injury and to enhance neuronal and glia survival, particularly oligodendrocytes, boosting functional recovery.^124,125^ On the other hand, Riluzole is a benzothiazole antiepileptic drug with the capacity to block hyperactivated voltage gated sodium channels and to regulate glutamatergic neurotransmission. These neuroprotective actions further limit the exacerbation of the TSCI inflammatory cascade by mitigating apoptosis and glutamate-induced excitotoxicity.^126^ Several studies, including clinical trials (ClinicalTrials.gov ID: NCT01597518),^127^ confirmed the capacity of this drug to spare neural tissue and boost beneficial neurological outcomes, even envisioning its use for the treatment of neuropathic pain.^128^ Other noteworthy clinical trials comprise the delivery of neuroprotector fibroblast growth factor (ClinicalTrials.gov ID: NCT01502631) and neuroregenerative agents such as anti-Nogo-A antibody (ClinicalTrials.gov ID: NCT03935321) and Cethrin/VX-210 (ClinicalTrials.gov ID: NCT02669849), investigating their capability to boost TSCI patients recovery by diminishing excitotoxicity or stimulating axonal sprouting, respectively.^129−131^ These neuroregenerative agents are responsible for enhancing neurite outgrowth and, consequently, rebuilding the neuronal network through different mechanisms. The monoclonal antibody anti-Nogo-A nullifies the activity of the CNS myelin inhibitor Nogo-A, while the Cethrin/VX-210 toxin blocks the repressive effect of CSPGs on neurons by interrupting the Rho-ROCK signaling pathway.

Overall, although each pharmacological agent is capable of reversing a specific pathological mechanism during secondary injury, the benefits of a single-target therapy are commonly suppressed by the complex set of overlapping biochemical events following TSCI. In this way, the development of multitherapeutic pharmacological strategies appears as an auspicious approach for combining various neuroprotective and neuroregenerative outcomes. However, intrinsic drawbacks such as the limited selectivity of the pharmacological agents, the negative side-effects triggered by collateral interactions between administrated drugs and the divergent therapeutic time windows of intervention need to be carefully addressed before implementation.^132^ Progress in this matter is, therefore, intimately associated with technological advances such as (1) the capability of advanced computer simulations to anticipate both pharmacokinetics and biodistribution of each pharmacological candidate; (2) the inclusion of smart drug delivery vehicles (e.g., graphene- or polymer-based nanocarriers) suitable for balancing their biodegradation profiles with temporally and spatially accurate drug deliveries; (3) the efficient application of adeno-associated viral (AAV) and nonviral vectors to directly introduce neurotrophic factors into neurons; and (4) the development of computer-based and biotechnological tools proficient to design and produce specific therapeutic constituents (e.g., biological compounds, drugs).^24,133−135^ For instance, in an advanced strategy including principles of genetic engineering, Leibinger *et al.*(136) successfully delivered AAV-hyper-interleukin-6 into the sensorimotor cortex of paralyzed adult mice (complete SCI model). The results showed a successful transneuronal transduction of motoneurons to other areas of the brain and through its rostrocaudal length, inducing a significant and sustainable regeneration of neuronal circuits (corticospinal and raphespinal tracts) that culminated in the locomotor recovery of both hindlimbs.

### GBMs for Biomolecules Delivery in the CNS

Scarce literature is currently available on the potential role that GBMs may assume as multifunctional nanocarriers for SCI therapeutics. Nevertheless, there is a real interest in converting the intrinsic structural and functional features of these nanomaterials into nontoxic drug delivery systems able to interface the damaged CNS (e.g., Alzheimer’s and Parkinson’s diseases, glioma).^9^ A prominent advantage is the large specific surface area provided by these nanomaterials, which enables superior interactions with a wide range of molecules, natural or synthetic, via either noncovalent (e.g., π-π staking, hydrogen bonding, electrostatic interactions) or covalent bonding. This encourages opportunities for engineering biocompatibility, solubility, surface chemistry, and biofunctionalization of GBMs to carry and/or hold therapeutic cargoes such as drugs, proteins, and genes.^137^

The presence of oxygen moieties on the surface of GO has placed this nanomaterial as a natural frontrunner to integrate drug delivery strategies, offering a customizable set of functionalization routes and, simultaneously, significant versatility to cross neural physiological barriers like the brain–blood barrier.^18,138^ For example, Song *et al.*(139) developed a GO nanocomposite combining both magnetic and molecular targeting by the coupling of superparamagnetic Fe_3_O_4_ nanoparticles and lactoferrin glycoprotein, correspondingly. This advanced nanocarrier, which could be guided under an external magnetic field, was then loaded with the anticancer drug doxorubicin hydrochloride (DOX) via strong π–π stacking with GO to enhance cell uptake, delivery capacity, and strong cytotoxicity with brain tumor cells *in vitro*. DOX was also loaded in a transferrin-conjugated PEGylated GO nanocarrier and tested in a rat model *in vivo*.^140^ Results showed that, after systemic administration, the transferrin functionalization enabled efficacious passage through the brain–blood barrier toward the tumor cells localized in the brain, where the nanocomposite inhibited glioma growth. Moreover, it was recently reported that the conjugation of GO nanosheets with both superparamagnetic iron oxide nanoparticles and the biocompatible polymer poly(lactic-*co*-glycolic acid) enhanced the circulation time of the loaded anticancer drug; precisely targeted the glioma by an external magnetic field; tracked the location of the nanocomposite via MRI; and efficiently released the drug *in vivo*.^141^

**Figure 5:**
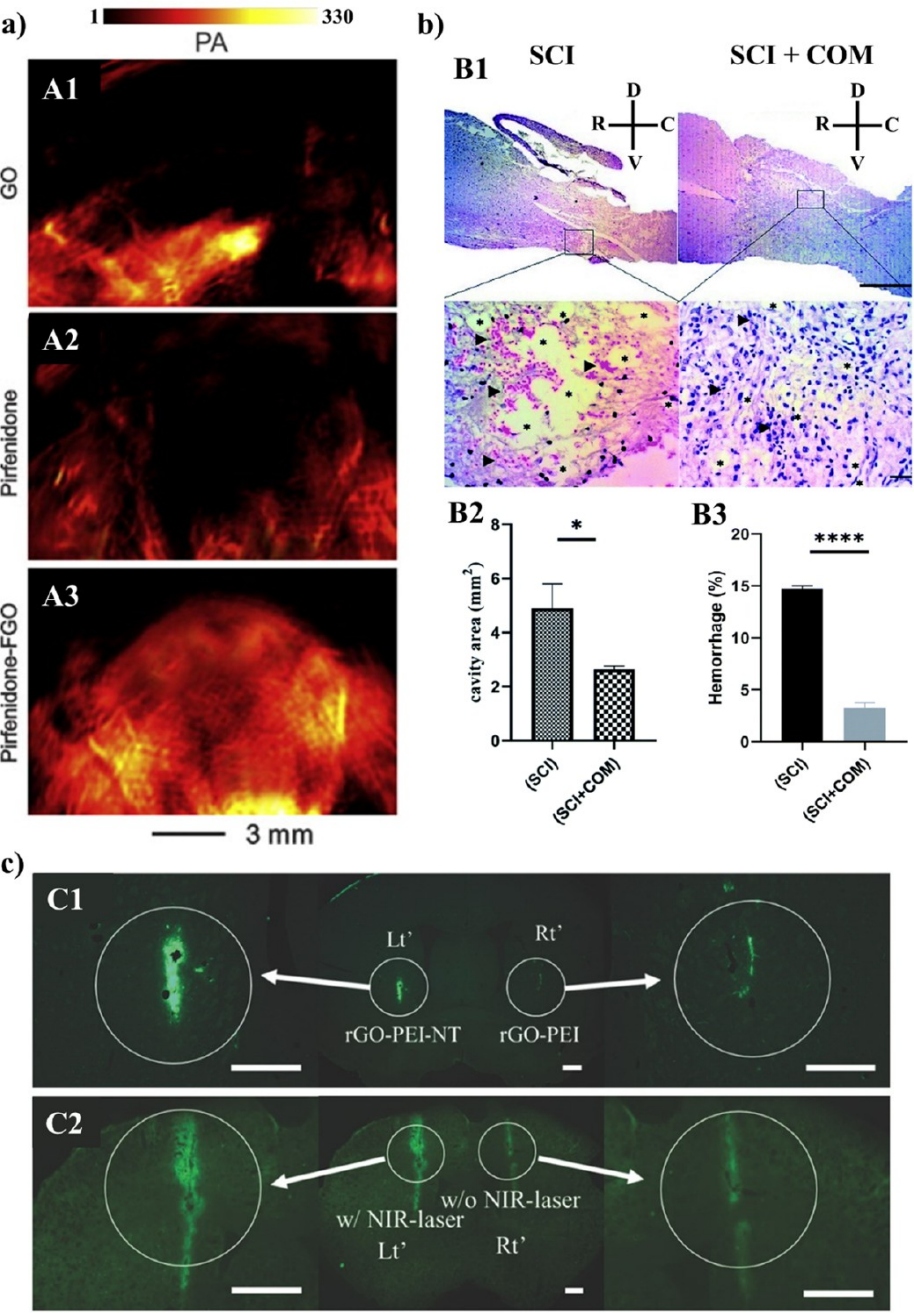
GBMs delivering biomolecules into the CNS. (a) Photoacoustic (PA) images of the cross-section of nude mice brain 8 h after tail vein injection of pure GO (A1), pirfenidone (**A2**) and pirfenidone-functionalized GO (FGO) (**A3**). Adapted with permission from ref (148). Copyright 2015 Elsevier. (b) Therapeutic effect of GO-PEG and GO-chitosan nanocomposites on SCI. (B1) Histological investigation of the spinal cord at 14 days postinjury by H&E staining at low magnification (4×), and high magnification (40×); higher magnification of the injury site obviously showed the cystic cavities (asterisks) and hemorrhage (arrowheads) in the SCI and treatment (SCI + COM) groups. (B2 and B3) Quantitative results for the cavity regions of sagittal segments and hemorrhage percentage at the lesion site in the spinal cord (*p < 0.05, ****p < 0.0001, *n* = 4 animals in each group). The scale bars are 1 mm (top) and 50 μm (zoom). Adapted with permission under a Creative Commons Attribution 3.0 Unported License from ref (149). Copyright 2021 Royal Society of Chemistry. (c) Frozen sections of C57BL/6 mice brains transfected with (**C1**) rGO- polyethylenimine-neurotensin/plasmid DNA (rGO-PEI-NT/pDNA) and rGO-PEI/pDNA, and (C2) rGO-PEI-NT with/without NIR laser irradiation. Scale bar = 600 μm. Adapted with permission from ref (154). Copyright 2016 Wiley-VCH.

The use of GO-based nanocarriers in the CNS is presently monopolized by cancer therapeutics because of the multitude of promising configurations able to surpass standard chemotherapy outputs, resulting in accurate delivery of drugs, proteins, and/or genes (e.g., microRNAs) while allowing, for example, bioimaging of the treatment progression in theranostic approaches.^142−145^ Based on this, GO-based nanocarriers hold the potential to counteract the neoplastic conditions responsible for provoking NTSCI (Chapter S1).^146^ In the case of TSCI, nanocarrier properties must be adjusted accordingly to the hostile conditions generated after the BSB rupture. For instance, Saxena *et al.*(147) demonstrated that polyethylene glycol (PEG)-coated liposomal nanocarriers with a size inferior to 200 nm were capable of extravasating into the lesion site during 96 h postinjury in a rat compression model. A large number of these nanoparticles were further phagocytized by reactive microglia after 1 week of inoculation. Another important issue relates to the possibility of GO-based nanocarriers to attenuate the hemorrhages that immediately follow SCI. To this regard, a study developed by Yang *et al.*(148) provided important guidelines relatively to workable functionalization routes of GO to successfully challenge CNS hemorrhages *in vivo*. Briefly, the carboxylic groups located on the GO surface were modified to better interact with the NH_2_ terminals of both the transcription activator peptide and methoxy PEG, leading to a functionalized nanocomposite with enhanced capability to cross the blood–brain barrier and efficiently circulate in the blood, respectively. After being loaded with pirfenidone, commonly used for CNS therapies, rats treated with these GO-nanocarriers presented less damage driven from the induced subarachnoid hemorrhage, further visualized by photoacoustic imaging due to the robust near-infrared (NIR) absorbance of GO (Figure 5a).

Alternatively, Yari-Ilkhchi *et al.*(149) recently studied the interface between GO-based nanocomposites and the injured spinal cord microenvironment. In this work, two alternative functionalization strategies were followed to fabricate two complementary therapeutic agents: GO-PEG and GO-chitosan, being GO bonded to PEG via 1-ethyl-3-(3-dimethyl aminopropyl) carbodiimide/*N*-hydroxysuccinimide (EDC/NHS) cross-linking, while chitosan was attached on GO via electrostatic interactions and hydrogen bonding. Interestingly, the simultaneous injection of both GO-PEG and GO-chitosan nanocomposites induced some neural recovery in a compression SCI model in rats. In detail, the performance *in vivo* of both nanocomposites prompted neuroprotective and anti-inflammatory outcomes including the reduction of cavity areas, hemorrhages, and scar formation (Figure 5b). The improvements in motor function were quantified by the 21-point Basso, Beattie, Bresnahan (BBB) locomotor scale with a score of 6 at 14 days postinjury. Comparatively, PEGylated graphene nanoribbons were successfully applied as fusogens to SCI rats (L1 contusion model), with a BBB score of 8 after 5 weeks postinjury accompanied by a low astrocytic reactivity and an enhanced axonal regrowth on the boundaries of the gray and white matters, respectively.^150^ Overall, these two pioneer reports pointed out that GBMs in the shape of nanocomposites can promote neuroprotective and neuroregenerative outcomes associated with functional recovery at the injured spinal cord. Furthermore, the absence of an accentuated microglia response encourages the exploration of advanced 3D GBM implants for neural regeneration.

However, there is still a long way ahead to further investigate the impact of these nanomaterials on the functionality of the spinal neural networks *in vivo*, including a multidisciplinary perspective for collecting and organizing all data resources able to accurately associate each GBM condition (e.g., dosage, physicochemical properties, functionalization routes) with specific *in vitro* and *in vivo* biological responses. For example, the small size (20–50 nm) of a GO-based T1MRI contrast agent was decisive to upgrade the tracking efficiency of labeled hMSCs within the brainstem in a rat model.^151^ Alternatively, GO-porphyrin nanocomposites with sharper edges and larger dimensions (276.1 nm) showed superior permeability through a brain–blood barrier *in vitro* model comparatively to their smaller (58.3 nm) counterparts.^152^ The comprehension of the underlying mechanisms governing the pharmacokinetics of each GBM is, unquestionably, a mandatory landmark for guaranteeing functional dynamics with CNS components, as analogous functionalization routes have been proven to trigger different responses both *in vitro* and *in vivo*. For example, standard PEG functionalization, which is often used to augment cytocompatibility, stability, and absorption of GBMs, could trigger counterintuitive outcomes for rGO nanosheets.^153^ In brief, the systemic injection of PEGylated-rGO in rats activated mechanisms of oxidative stress-mediated damage responsible for stimulating astrocyte dysfunction and, therefore, severely compromising the integrity of the blood–brain barrier at the hippocampus.

Even though GO is currently the preferential building block for developing advanced nanocarriers and nanocomposites for CNS therapeutics, some reports have already validated rGO nanosheets as a promising alternative to deliver neuroprotective and/or neuroregenerative biomolecules. For example, Hsieh *et al.*(154) proposed a multiresponsive, nonviral rGO-based nanocarrier to perform gene transfection to neurons in a rat brain model. In this study, rGO was first functionalized with polyethylenimine via electrostatic interactions to optimize the loading and delivery efficiencies of the nanoplatform, hen being cross-linked to the neuron-specific factor neurotensin to enhance cytocompatibility and drive specific neuronal targeting. The DNA plasmid was also attached via electrostatic interactions, and its delivery to neurons was optimized by a NIR stimuli sequence (Figure 5c). Briefly, NIR irradiation was crucial not only to adjust the permeability of the cell membrane and guarantee an appropriate nanocarrier uptake, but also to allow an optimized release from the endo/lysosome entrapment. It is important to note that NIR stimulation also proved to interfere with the brain–blood barrier permeability to enhance the passage of biomolecules,^155^ thus anticipating the possibility of controlling the entrance of therapeutic agents through the BSB in a similar fashion. Such intervention may possibly broaden the range of GBM nanocarriers and nanocomposites to be applied in SCI repair since NIR external stimuli could diminish the endogenous barrier effect on nanomaterials delivery. In this sense, there are encouraging advantages in the exploration of distinctive responsiveness of GBMs to NIR irradiation for treating SCI. For instance, the NIR-induced photothermal effect on GBMs is proficient to trigger an accurate desorption of loaded drugs from the nanocarrier.^156^ Also, NIR irradiation presents a highly functional deeper tissue penetration capacity that enables an efficacious detection of GBMs *in vivo*.^157^

Regarding GQD-based nanocomposites, the spotlight is not exclusively placed on functionalization approaches to enhance cell-nanomaterial interactions while maintaining the photoluminescence properties responsible for NSCs tracking.^158^ Indeed, as recently pointed out by Xiao *et al.*,^159^ GQDs present an enhanced set of features (e.g., large surface area, active functional groups, solubility) that can be successfully used to carry therapeutic biomolecules to the damaged CNS. In this particular case, GQDs were conjugated with the neuroprotective peptide glycine-proline-glutamate via EDC/NHS coupling with the purpose of limiting the progression of Alzheimer’s disease in a rat model. This nanocomposite was capable of binding to the Aβ monomer and therefore inhibiting the aggregation of Aβ1–42 fibrils in the brain, leading to a significant moderation of the typical Alzheimer’s pathophysiological outcomes such as neuroinflammation and neurodegeneration.

## The Promise of GBMs to Record and Stimulate Neural Circuits

### Neuromodulation and Robotics for SCI Therapeutics

There is a growing interest in instituting neuromodulation as a superior SCI therapeutic modality considering the potential of electrical stimulation to enhance and/or modify neuronal activity at the spinal cord–spinal cord stimulation (SCS)—or in the supraspinal circuits—brain stimulation—toward functional recovery.^160^ Relative to SCS, the location and invasiveness of the applied electrodes, as well as the specifications of the delivered stimulation (e.g., frequency), can be adapted to match specific therapeutic requirements. Epidural spinal stimulation (ESS), which involves the surgical implementation of a set of electrodes, is the most well-known SCS technique due to its significant impact on managing chronic pain.^161^ The use of this approach for regenerating spinal cord neural networks after TSCI has been studied in the past few years, highlighting the successful recovery of both voluntary (e.g., upper and lower limbs) and involuntary (e.g., bowel and lower urinary) movements.^162−164^ In this sense, a recent clinical trial (ClinicalTrials.gov ID: NCT03026816; Table S1) have shown that patients suffering from motor complete TSCI were able to regain spontaneous volitional movements after long-term ESS treatment.^165^ More recently, Courtine and co-workers^166^ have further upgraded this ESS approach with an array of electrodes precisely targeting the dorsal roots involved in leg and lower-trunk movements (ClinicalTrials.gov ID: NCT02936453). Accompanied by biomimetic activity-specific stimulation programs conducted during neurorehabilitation (5 months), complete paralyzed patients were able to perform complex motor functions such as standing, walking (within 1 day of stimulation), cycling, swimming, and controlling trunk movements.

The neural plasticity of the spinal cord can be also modulated via invasive intraspinal macrostimulation (i.e., electrodes are incorporated within the spinal cord) and noninvasive transcutaneous SCS (i.e., electrodes implanted on the skin above the vertebral column). Although such techniques differ in accuracy (superior in intraspinal macrostimulation) and long-term biocompatibility (superior in transcutaneous SCS), their findings using animal models and clinical trials (ClinicalTrials.gov ID: NCT03998527) confirm promising neurological recoveries after TSCI, especially when combined with other therapeutic approaches.^167,168^ Complementary to SCS, the application of brain stimulation (e.g., transcranial direct current stimulation (tDCS) and transcranial magnetic stimulation) to TSCI patients with residual ascending/descending pathways has provided encouraging functional outcomes.^169^ Indeed, the validation of brain stimulation as a trigger for lower extremity function is currently being evaluated by a clinical trial (ClinicalTrials.gov ID: NCT03237234). In this trial, a randomized group of TSCI patients receive tDCS during motor skill training exercises with the purpose of analyzing variations in walking function, spasticity, and ankle strength over time. Moreover, the association of transcranial magnetic stimulation and specific training exercises has been proven to induce the enhancement of supraspinal neural plasticity in TSCI patients, improving the motor function of their hands and legs.^170,171^

Advances in neuromodulation are substantially supporting the development of brain–-computer/machine interfaces (BCI) proficient to directly use the acquisition and translation of neuronal activity signals to generate motor commands in an external device, such as a computer cursor, a wheelchair, or a robotic prosthesis.^172,173^ In this context, findings reported by an ongoing clinical trial (ClinicalTrials.gov ID: NCT02550522) demonstrated that a tetraplegic patient was able to successfully control a whole-body exoskeleton, which was connected via wireless to a surgically implanted cortical electrode array responsible for recording epidural electrocorticographic signals.^174,175^ At the end of the training stage, these studies included the performance of bimanual tasks using eight degrees of freedom and a suspended walking capability that endured for 24 months after implementation. Another important modality of BCI intends to directly bypass the injured spinal cord by transmitting electrical pulses between the brain and the denervated effectors (e.g., muscles) with the purpose of establishing an alternative communication route for sensory and/or motor information. For example, a clinical trial (ClinicalTrials.gov ID: NCT01997125) has already confirmed that the motor cortex activity of a TCSI patient could be recorded using an invasive intracortical microelectrode array in order to be continuously decoded and transmitted to a neuromuscular electrical stimulator located on the participant’s arm.^176^ This successful translation of neuronal information allowed the patient to perform complex movements in real time such as hand motion and object manipulation. Complementarily, another clinical study (ClinicalTrials.gov ID: NCT01894802) highlighted the potential of using intracortical macrostimulation of the somatosensory cortex to mimic conventional sensory inputs, enabling the tetraplegic participant to recognize and localize pressure-like stimulus that were applied into a prosthetic hand.^177^ Promising results obtained from brain–spine interfaces to drastically augment the mobility of injured rats and primates are also noteworthy.^178,179^ In these cases, the decoded motor cortex activity was used to generate specific ESS patterns that were then delivered below the lesion site with the purpose of triggering voluntary locomotor movements.

Notwithstanding the potential shown by neuromodulation and robotics to boost approaches capable of either augmenting/replacing the sensorimotor functions of TSCI patients or assisting them during rehabilitation training, the developed technology is not yet able to (1) flawlessly acquire and process neuronal signals; (2) ensure affordability and long-term durability; (3) reach efficacy levels capable of scaling the auspicious individual case reports; and (4) guarantee a feasible adaptation to the existing healthcare infrastructure and personnel.^160,180^ Such challenges demand a synergetic contribution of several research fields (e.g., computer engineering, material science, medicine) in order to combine the technological and material advances with an accurate understanding of the underlying SCI mechanisms, leading to the design and development of more robust and large-scale clinical studies.

### Graphene-based Electrodes Interfacing the CNS

Presently, metal-based electrodes (e.g., Ag, Au, Pt, stainless steel) are considered to be standard platforms to stimulate and/or record the neuronal activity of neural tissues due to their stability and easy manufacturing. However, their performance *in vivo* is often compromised by biocompatibility and impedance issues, two important limitations that the incorporation of nontoxic polymers (e.g., silicon) is able to attenuate but not completely overcome.^181^ Indeed, as rigid neural electrodes present low flexibility and incompatible mechanical compliance relative to characteristically soft CNS elements, their long-term implantation typically prompts foreign-body responses that severely diminish recording/stimulation outputs.^182^ In this context, Minev *et al.*(183) developed a soft, transparent, and multimodal electrode for resembling both morphological and mechanical properties of the dura mater. Due to its features, this serpentine-like electrode was feasibly implanted within the spinal subdural region (covering L2 to S1 segments) in a rat model with the purpose of activating locomotor neuronal circuits below the contused spinal cord segment (T8). Interestingly, rats stimulated with soft electrodes performed complex motor activities during 6 weeks after implantation without signs of neuroinflammation. On the contrary, stiffer electrodes provoked a local deformation of the spinal cord that the accentuated the presence of microglia and astrocytes and deteriorate motor performance as soon as 1–2 weeks postinjury. Taking this into account, mechanical mimicry (in the order of a few kPa, or even lower) must be a key design criterion for electrode systems targeting SCI. It is important to notice that neural tissues typically soften after injury and their endogenous mechanical properties differ according to anatomical features (e.g., white vs gray matters, dura mater).^184−186^ So, an accurate biomechanical evaluation of the targeted location and adjacent areas should be considered mandatory prior to electrode implantation in order to prevent tissue damage by mechanical mismatch, the withdrawal of neighboring neurons in the electrode surroundings due to tissue swelling and aggressive activation of local astrocyte/microglia toward the formation of a scar tissue encapsulating the probe.^187,188^ In the long-term, as the distance between the electrode and neuronal cell bodies must not be greater than100 μm for guaranteeing a stable recording, sparse neurons together with the scar barrier effect decisively contributes to isolate the probe from the tissue and increase its electrical impedance.^189^

Alternatively, or in combination with metal electrodes, the next generation of flexible and stretchable electronics is being robustly fueled by graphene, touching many scientific areas from energy-harvesting to tactile sensors and bioinspired platforms.^190^ For example, the unique mechanical properties of graphene^191^ enabled the fabrication of omnidirectionally stretchable electrodes capable of maintaining a high electrical reliability under a wide range of deformation conditions like bending, buckling, folding, and stretching.^192^ Such performance has the potential to narrow the mechanical mismatch between neural electrodes and the CNS, guaranteeing, for instance, the absence of undesirable local compression during spinal cord bending, as well as a suitable elongation during tissue stretching in native behavioral movements of the animal/person. The biological and mechanical integration of electrodes into neural tissues can be also optimized by upgrading their designs from planar devices to 3D macroporous nanoelectronic scaffolds, which could be further accommodate polymers and, consequently, generate highly viable cellular microenvironments in combination with excellent electronic properties.^193^

**Figure 6:**
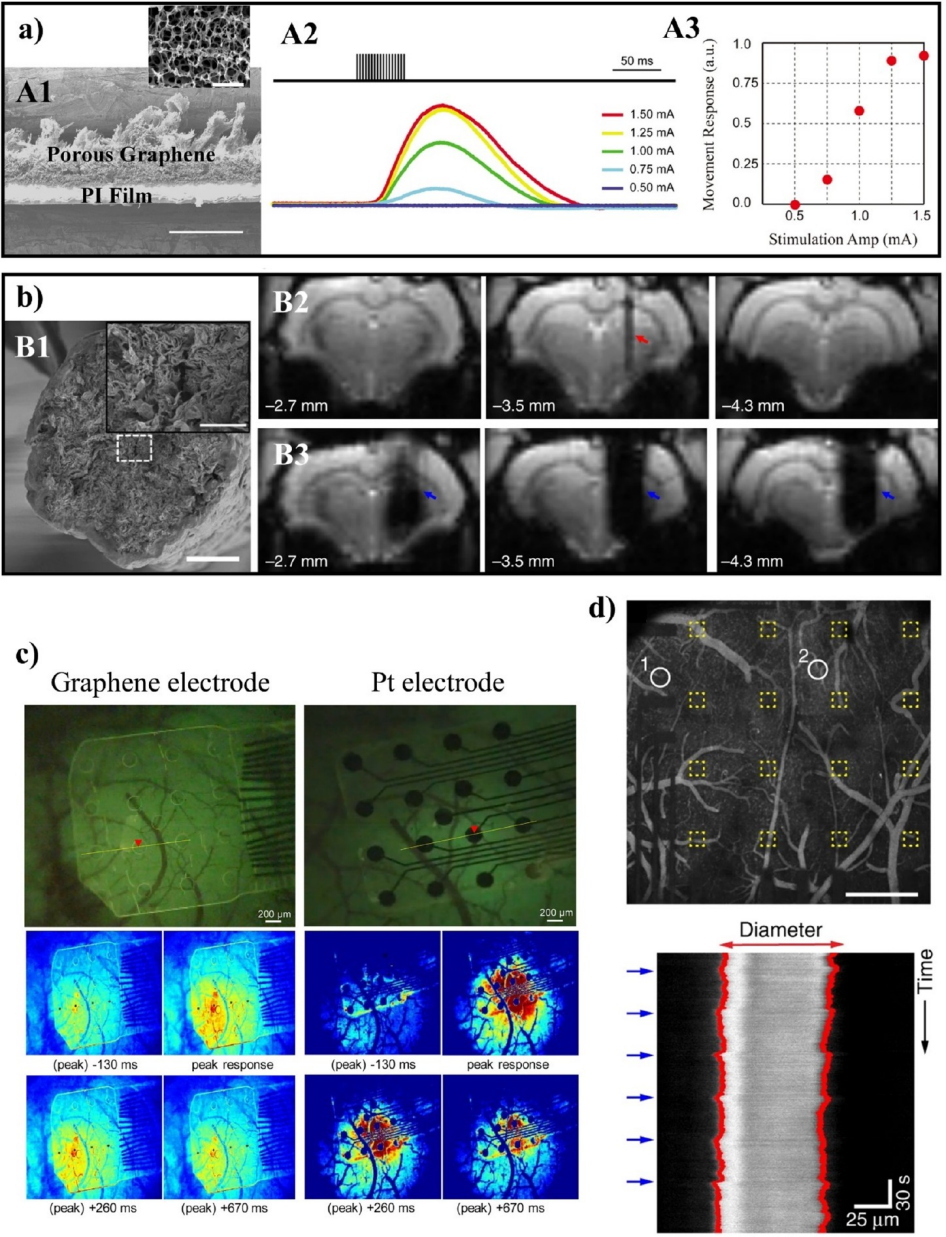
Graphene-based electrodes interfacing the CNS. (a) *In vivo* stimulation of cortical tissue by a 3D porous graphene electrode. (A1) Electrode morphology. Scale bars = 100 and 2 μm (inset). (A2) Stimulus evoking current (representing movement) response of the flex sensor in arbitrary units. (A3) Movement response versus stimulation amplitude. Adapted with permission under a Creative Commons Attribution 4.0 International License from ref (194). Copyright 2016 Springer Nature. (b) *In vivo* assessment of MRI artifacts after electrode implantation. (B1) Electrode morphology. Scale bars = 20 and 5 μm (inset). Representative three serial coronal scans from rostral (left) to caudal (right) of echo-planar images from rat brains with 3D microfibers graphene (B2) and PtIr (B3) bipolar microelectrodes (middle images depicting electrode implant sites). The numbers in each image denote the relative distance from bregma. Adapted with permission under a Creative Commons Attribution 4.0 International License from ref (201). Copyright 2020 Springer Nature. (c) Cortex electrical stimulation through microelectrocorticography electrodes and corresponding neural activity in GCaMP6f mice. (Top) Visualization of the fluorescence neural response after stimulation with a single graphene electrode site (marked with a red triangle) and a single Pt electrode site (marked with a red triangle). (Bottom) Visualization of the intensity of neural responses to 100 μA of electrical stimulation at times −130 to +670 ms of peak response with the graphene electrode array (left) and 500 μA of electrical stimulation at similar times with the Pt electrode array (right). Adapted with permission from ref (206). Copyright 2018 American Chemical Society. (d) Measurement of vascular responses to optogenetic photostimulation below graphene microelectrode arrays in Thy1-ChR2 mice. (Top) Location of diving arterioles for diameter measurements with 2-photon microscopy imaging. Yellow outlines indicate single graphene electrodes. Data were acquired after intravascular injection of FITC-dextran (2 MDa). Scale bar = 500 μm. (Bottom) Line-scan mode was used to measure time courses of single arteriole diameters. Blue arrows indicate delivery of 473 nm laser stimuli, and red lines indicate computed vessel borders used for estimation of diameter changes. Adapted with permission under a Creative Commons Attribution 4.0 International License from ref (209). Copyright 2018 Springer Nature.

Regarding electrical performance, Lu *et al.*(194) evaluated flexible 3D porous graphene electrodes (pore size of 0.2 μm) interfacing the brain surface in a rat model. Their optimized effective surface area was decisive to enhance charge injection capacity (CIC) to 3.1 mC/cm^2^. This electrode was able not only to efficiently record physiological oscillations and low amplitude evoked potentials from the somatosensory cortex but also to conduct minimally invasive cortical stimulation for triggering leg movements with great precision (Figure 6a). In another study, Apollo *et al.*(195) fabricated 3D brush-like graphene electrodes (microfibers diameter of 40–50 μm) able to present charge injection levels of tens of mC/cm^2^ (from 14 to 62 mC/cm^2^, depending on the electrochemical impedance spectroscopy model) to successfully stimulate retinal ganglion cells *in vitro*. Results also showed that, after the degradation of the sucrose coating *in vivo*, the rGO electrode was capable of recording brain activity for 15 min in a feline model. Generally, the CIC values reported for graphene-based electrodes are comparable, if not superior, to other advanced neural platforms such as Pt nanoporous systems (3 mC/cm^2^),^196^ CNT-based microfibers (6.5 mC/cm^2^)^197^ and poly(3,4-ethylene dioxythiophene)-based microelectrodes (2.9 mC/cm^2^),^198^ proving their competitive behavior to stimulate CNS tissues. Despite this encouraging competence, to effectively interact with the spinal cord, graphene electrodes should be able to reach suggested levels of charge density (e.g., >30 mC/cm^2^ for chronic pain treatment) and to conjugate superior performance with the approved safety threshold to prevent tissue damage (e.g., neurotoxicity, electroporation).^199^

The therapeutic relevance of graphene-based electrodes is not exclusively dependent on ensuring superior CIC levels but also on their inherent low impedance and high signal-to-noise ratio, being possible to optimize such features by functionalization. For instance, Wang *et al.*(200) sequentially coated a rGO microfiber (diameter of 20 μm) with Pt and parylene-C with the purpose of decreasing the resistivity and enhancing the biocompatibility and robustness/flexibility of the composed electrode, respectively. In fact, the final construct presented a superior set of optimized properties such as a notable CIC (10.5 mC/cm^2^), a high surface area (169 μm^2^) provided by the porous rGO component, a diminished specific impedance (1.9 MΩ μm^2^ at 1 kHz) comparatively to other reported electrodes (e.g., Pt = 390 MΩ μm^2^ at 1 kHz and CNT = 20.4 MΩ μm^2^ at 1 kHz),^196,197^ and an enhanced signal-to-noise ratio *in vivo* (9.2 dB for a single microelectrode) when recording the cortical neural activity in a rat model. A comparable rGO microfiber electrode (diameter = 75 μm; CIC = 10.1 mC/cm^2^; specific impedance = 15.1 MΩ μm^2^ at 1 kHz) was developed by Zhao *et al.*(201) to conduct deep brain stimulation in a rat model of Parkinson’s disease without compromising the performance of functional MRI applied simultaneously. Results highlighted the ability of graphene-based electrodes to prevent MRI artifacts (Figure 6b) and, subsequently, to allow an accurate mapping of brain activity during stimulation, which was not achievable by traditional metal-based devices. Conceptually, a proper adaptation of such graphene-mediated stimulation-imaging synergy could contribute to a more robust evaluation of the modulatory effects triggered by tDCS or SCS in SCI patients. This could allow, for instance, an enhanced detection of variations in the blood-oxygenation-level-dependent responses of the spinal cord^202^ and the precise detection of active brain–spinal cord circuits.^203^ Moreover, graphene-based electrodes may be more advantageous in combination with MRI at the spinal cord due to their high performance and their ability to significantly prevent artifacts. Considering that the limitations of spinal cord MRI are majorly related to susceptibility-induced artifacts (e.g., provoked by bones), elevated physiological noise, and singular neural/vascular organization,^204^ the implanted electrode must be capable of minimizing any substantial magnetic field interference deteriorating image acquisition.

Alternatively to graphene-based electrodes with a 3D configuration tendentially offering a better electrical response, planar platforms based on monolayered graphene present optical properties very appealing for neuroimaging. For example, Kuzum *et al.*(205) developed a transparent and flexible graphene electrode capable of performing simultaneous electrophysiological recording and calcium imaging of hippocampal slices *in vitro* without producing light-induced artifacts. When challenged *in vivo*, this graphene electrode successfully recorded brain activity in a rat model, revealing a superior performance than Au electrodes due to a six-fold higher signal-to-noise ratio. More recently, Park *et al.*(206) used a transparent graphene electrode to perform simultaneous electrical stimulation and optical monitoring *in vivo*. Results showed that it was possible to accomplish a full-field view of the area beneath the electrode and, therefore, follow the resultant brain spatiotemporal dynamics by using transgenic mice (Figure 6c). Furthermore, the authors identified cathode leading stimulation as a more efficient modality than anode leading stimulation, considering the higher fluorescent intensity of the stimulated neurons as well as their faster response to the stimuli. Considering CNS therapies, the wide spectrum transparency displayed by this class of graphene electrodes, which goes from UV to IR, offers important advantages relative to common opaque metal electrodes such as (1) visualization of the immediate and long-term effects triggered by electrical stimulation on neural cells; (2) monitorization of immune responses and progression of scar tissue in both targeted and neighboring areas of the implanted electrode; and (3) development of multimodal therapeutic devices able to combine cell imaging and monitoring of neuronal activity with electrical and/or optical stimulation patterns.^207^

Transparent graphene electrodes provide an augmented area of optically accessible tissues, enabling efficient full field optogenetic stimulation and, subsequently, a light transmittance level able to guarantee enhanced *in vivo* imaging (e.g., cortex and cerebral vasculature via fluorescence microscopy).^208^ For example, the graphene electrode fabricated by Thunemann *et al.*(209) enabled optogenetic stimulation and 2-photon microscopy while recording the cortical activity in a transgenic mice model. The acquisition of local field potentials together with the analysis of calcium transients could be valuable tools to characterize spiking neuronal circuits *in vivo*, complementing the modulation of neurovascular responses (e.g., arteriolar dilatation, cortical hemodynamics) triggered by stimulated neurons (Figure 6d). Although the inclusion of graphene electrodes into optogenetics for CNS neuromodulation is currently limited to brain tissue, the possibility of recording neuronal activity from stimulated areas could serve to unmask and control any remaining viable sensory/motor circuits at the injured spinal cord. It is therefore expected that implantable optogenetic platforms could soon include transparent graphene electrodes near light sources (e.g., light emitting diode arrays) to guarantee simultaneous control over the stimulation/reading of the selected neural area. The upgrade of the electronic functionalities of the implantable optogenetic devices with graphene electrodes is, indeed, highly feasible considering the matching requirements: flexibility, soft mechanical properties, and a miniaturized wireless system.^210−212^

## The Promise of GBMs to Bridge the Injured Spinal Cord

### Biomaterials for SCI Therapeutics

Biomaterials are undoubtedly the most significant cornerstones for developing advanced approaches for the treatment of SCI. Their preponderant impact is not only related to the direct support of neural cells *in vivo* but also to their capability of intersecting many neuroprotective and neuroregenerative routes (e.g., bioelectronics, cell and drug deliveries) toward more complex and efficient therapeutic strategies.^23^ Commonly, biomaterials are obtained from either natural or synthetic polymers, or their combination, leading to an enormous array of possible biocompatibility and biodegradability profiles. On the one hand, natural polymers (e.g., agarose, alginate, chitosan, collagen, HA) are especially attractive to promote biomimetic cellular responses because of their essentially biocompatible nature and intrinsic binding sites. For example, the biopolymer HA, which specifically inherits fundamental properties of the spinal cord ECM, appears to be a frontrunner candidate to rebuild the SCI microenvironment due to its capability of recreating cell–material interactions that resemble the healthy neural tissue.^213^ Conversely, although collagen is a very popular natural polymer for SCI applications,^214^ its selection could be counterintuitive considering its scarceness in the healthy spinal cord and its abundance in the scar tissue after injury. Nonetheless, natural polymers are limited by poor mechanical properties, reduced structural integrity, and accentuated pH and thermal sensitivities. The application of this class of polymers is also restricted by the lack of available processing methodologies and their inherent low reproducibility derived from the significant variability associated with their source (e.g., batch to batch inconsistency). On the other hand, synthetic polymers, such as polycaprolactone (PCL), PEG, polylactic acid (PLA), and poly(lactic-*co*-glycolic acid) (PLGA), have a consistent source of raw materials and balance their higher risk of immune responses with a versatile engineering of their physicochemical properties (e.g., easy biochemical functionalization and sterilization). This feasibility is further translated into cheaper and more reproducible processing techniques, as well as superior design flexibility.

To expand their therapeutic scope, biomaterials can be (1) shaped by microfabrication techniques in order to acquire specific architectures (e.g., porous, fibrous) suitable for scaffolding the injured spinal cord in a more accurate fashion; (2) functionalized with biomolecules to enhance biocompatibility and/or stimulate specific neural cell responses (e.g., adhesion, differentiation, proliferation) and (3) seeded with cells to deliver them into the SCI or to produce a functional tissue-like structure prior to implantation.^215^ The synergetic combination of these options gives rise to complex TE strategies able to induce neuroprotective mechanisms, based on the accurate delivery of pharmacological agents or cells, and/or neuroregenerative pathways, proficient to bridge the lesion for directly supporting the growth of healthy tissue.

Over the years, findings reported from animal models have persistently encouraged the application of TE scaffolds in SCI therapeutics, leading to the recent translation to clinical trials. Indeed, two interrelated studies are currently evaluating the safety and therapeutic effect of a PLGA porous scaffold functionalized with poly(l-lysine) (PLL)—“Neuro-Spinal scaffold”^216,217^—into TSCI patients with an initial neurological level of T2–T12 and AIS A classification. Results confirmed the safety of the procedure and, importantly, highlighted that 7 patients (out of 16) improved their neurological status to AIS B (*n* = 5) or AIS C (*n* = 2) after 6 months postimplantation (ClinicalTrials.gov ID: NCT02138110; Table S1).^218^ These promising outcomes are being reinforced in a clinical trial comparing the responses of TSCI patients treated with the Neuro-Spinal scaffold with those treated with standard-of-care open spine surgery (ClinicalTrials.gov ID: NCT03762655). Alternatively, there are ongoing clinical trials investigating combinatorial scaffolding approaches as that for a linear ordered collagen scaffold named NeuroRegen scaffold, responsible for bridging the SCI area and delivering bone marrow mononuclear cells (BMMCs), MSCs and NSCs (ClinicalTrials.gov IDs: NCT02352077; NCT02688062; NCT02510365 and NCT02688049). In these trials, impressive sensory and motor recoveries were documented in two TSCI patients, who were treated with a NeuroRegen scaffold seeded with MSCs, showing improvements in their neurological status from complete AIS A to incomplete AIS C after 12 months postimplantation.^219^ Divergently, the implantation of a NeuroRegen scaffold seeded with BMMCs, although proven to be a safe procedure, was not able to induce a significant functional recovery in the tested TSCI patients during a 3 year follow-up period.^220^ Finally, a collagen scaffold is also being tested together with the application of epidural electrical stimulation, evaluating their combinatorial therapeutic effects in both acute and chronic TSCI patients (ClinicalTrials.gov ID: NCT03966794).

It is important to note that the development of human trials investigating scaffolds for spinal cord TE applications is still in its infancy. Indeed, these ongoing studies mostly address the safety and validation of the scaffold transplantation process, giving, nevertheless, preliminary insights into the resultant neuroregenerative outcomes. The tested scaffolds present simple designs and compositions (e.g., collagen, PLGA), in contrast to the heterogeneity of the lesion geometry and the complex array of overlapping biochemical events that follows TSCI. At the preclinical stage, numerous scaffolding approaches are being continuously developed and tested both *in vitro* and *in vivo* with the purpose of unveiling multifaceted TE strategies to efficiently repair the injured spinal cord. The most promising advances reported during the past few years rely on multifunctional designing concepts for bridging the injury area and the combination of distinctive biomaterials with complementary functionalities.^221^

### Proof-of-Concept in 2D Systems

The efficient growth of high-quality monolayer/few-layered graphene via CVD and its subsequent easy transference to arbitrary substrates have decisively boosted the large-scale production of 2D graphene-based biomedical platforms, particularly electrodes.^222^ Indeed, although such substrates are theoretically ideal components for biosensing and microelectronics, they could also support neural cell cultures with the purpose of either promoting meticulous studies *in vitro* or expanding specific cell lineages in a controlled fashion before transplantation. Under the scope of SCI therapeutics, it is important to comprehend and adjust the interface between graphene-based constructs and neural cells, including key structural and functional dynamics related to synaptic transmission, variations in membrane biophysics, neurite elongation, and differentiation.

**Figure 7:**
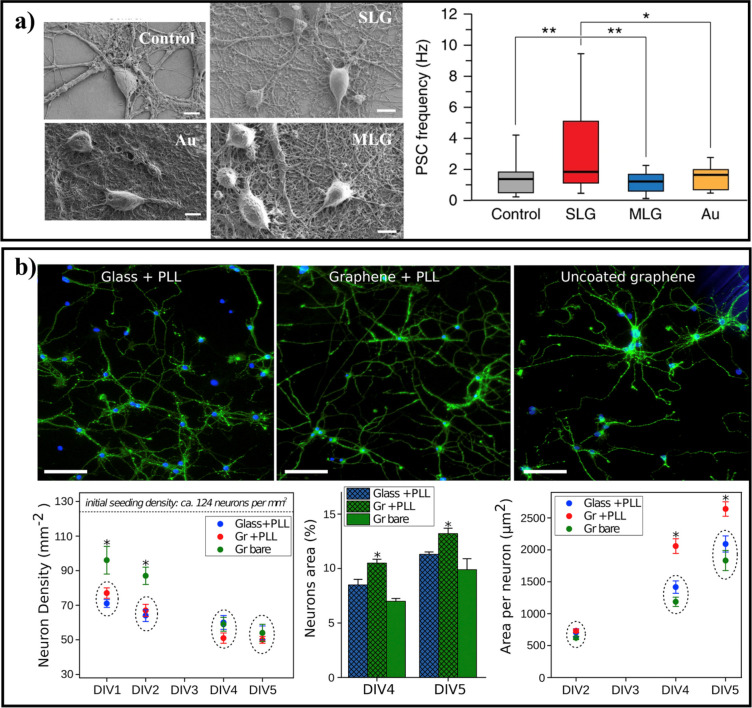
Neural cells onto 2D graphene-based substrates. (a) Hippocampal neurons cultured onto 2D graphene-based substrates. Representative SEM micrographs highlighting neurons morphology after 10 days *in vitro* on control (glass), gold (Au), single-layer graphene (SLG), and multilayer graphene (MLG) substrates (left). Scale bars = 10 μm. Box plot summarizing the frequency values of the postsynaptic currents measured for the different substrates (right). Adapted with permission from ref (223). Copyright 2018, Springer Nature. (b) Primary mouse embryos hippocampal neurons interfacing 2D graphene- and PLL-based substrates. Immunofluorescence image of neurons grown on PLL-coated glass (glass + PLL), PLL and graphene-coated glass (Gr + PLL) and graphene-coated glass (Gr bare) at 5 days *in vitro* (top). Dapi (blue) and synapsin (green) labeling are superimposed. Scale bars = 100 μm. Quantitative analysis of neurons density as a function of the incubation time (bottom, left); the area fraction covered by neurons (bottom, center) and the area covered by individual neurons on pristine graphene, coated glass, and graphene samples (bottom, right). Reproduced with permission from ref (229). Copyright 2016 Elsevier.

The development of neuronal cultures onto pristine graphene monolayers commonly results in the generation of viable and highly interconnected networks, presenting mature neurons with expanded neurite arbors for synaptic activity. Interestingly, these graphene films, comparative to 2D multilayer substrates, do not promote substantial differences in the spatial rearrangement nor in the number of synaptic connections, being responsible, nevertheless, for a significant increase in neurotransmission frequency due to alterations in the homeostasis of neuronal membrane currents. As a matter of fact, the efficient adsorption of K^+^ ions by single-layer graphene substrates proved to limit the extracellular availability of these ions and, therefore, intensify the excitability of the cultured neurons, leading to the augmentation of the frequency of postsynaptic currents without altering their amplitude (Figure 7a).^223^ The molecular machinery of the neuronal membrane is also altered when contacting a graphene monolayer film, resulting in a local increase of cholesterol levels that boosts the production of synaptic vesicles as well as augments the probability of their release.^224^ In a particular study, Convertino *et al.*(225) have recently reported that a 2D graphene film was capable of promoting a sustainable axonal elongation during 3 days of neuronal culture by successfully interfering with the retrograde transportation of nerve growth factor vesicles from the axon to the soma. Similarly, 2D graphene films fabricated via CVD supported the adhesion and proliferation of NSCs, which presented a healthy morphology with filopodia strongly interacting with the substrate.^226^ As a result, the resting potential of the cultured NSCs became more negative, and their maturation was accelerated toward functional neurons (preferentially), astrocytes, and oligodendrocytes. These promising results were also found for other graphene derivatives such as 2D rGO films, where NSCs also differentiated into those three neural cell types and generated functional neural networks *in vitro*.^227^

The impact of 2D graphene films on the behavior of neural cells can be modified according to the characteristics of the specific substrate while it will be transferred. For example, a monolayer of graphene placed on top of standard culture matrixes such as laminin and poly-d-lysine did not camouflage the inherent bioactivity of these substrates, preserving cell viability and axonal outgrowth in comparison to uncoated platforms.^228^ However, results also showed that, due to the direct contact with graphene, the cation channel activity of cultured neurons increased, notwithstanding the reduction of voltage-gated Na^+^ and K^+^ channels. It is often common to functionalize the surface of 2D graphene-based substrates with PLL in order to enhance the attachment, spreading, and growth of neural cells, as well as to optimize the generation of interconnected neuronal networks with a high density of elongated neurites suitable for boosting synaptic activity (Figure 7b).^229,230^ The hydrogenation of graphene via remote hydrogen plasma treatment was also recently pointed out as an efficient methodology to modulate neuronal responses *in vitro*.^231^ Comparatively to pristine graphene, the hydrogenated graphene guaranteed a more hydrophilic surface for cultured neurons, prompting an enhanced cell–material interface responsible for modulating cell morphology (homogeneous distribution vs clusters and aggregates) and neuronal activity (enhanced excitatory synaptic connections and miniature postsynaptic currents).

Alternative strategies have evidenced that adding biomolecules and chemical groups onto the oxygenated surface of GO could lead to the efficient manipulation of neurons and NSCs *in vitro*. For instance, it was reported that a GO substrate functionalized with amino groups promoted superior neurite branching and outgrowth comparatively to neurons cultured onto GO films with more negatively charged surfaces.^232^ Following this trend, Weaver and Cui^233^ successfully cross-linked interferon-γ and platelet-derived growth factor onto the surface of a GO-poly(3,4-ethylenedioxythiophene) nanocomposite film with the purpose of directing the differentiation of NSCs into either neurons or oligodendrocytes, respectively. Another study highlighted that the uniform distribution of graphene nanoplatelets in a chitosan matrix promoted efficient hydrophobic interactions with the ECM proteins, leading to a superior adsorption capability relatively to chitosan and chitosan-MWCNT films.^234^ Consequently, cultured hippocampal neurons were able to better adhere and proliferate into chitosan-graphene substrates, where their neurites extended radially in opposition to the elongated cell morphology induced by the chitosan-MWCNT composition. The response of astrocytes when cultured on a GO substrate functionalized with synthetic phospholipids was evaluated by Durso *et al.*,^235^ who described increased cell adhesion relatively to nonfunctionalized GO and rGO platforms without evidencing signs of gliotic reaction.

In addition to surface functionalization approaches, 2D graphene-based substrates used to deliver customized electrical and optical patterns have also shown potential to mediate specific neural cell responses, including NSCs differentiation.^14,236^ The opportunities offered by such electrical responsive platforms are mostly associated with the ideal principle of combining neuromodulation standards (e.g., SCS) with cell-based therapies *in vivo*, the electrical stimulation being the trigger for pursued cell processes such as differentiation and migration. In this regard, numerous *in vitro* studies have investigated strategies for tailoring the excellent electrical coupling between 2D graphene materials and neurons. For example, Park *et al.*(237) proved the ability of graphene films to enhance the Ca^2+^ related activity of differentiated neurons when electrical stimuli were applied. As a matter of fact, by adjusting the concentration of intracellular Ca^2+^, external electrical cues proved to be proficient to influence critical events of the neuronal circuits such as excitability and transmission of neuronal signals.^238^ Likewise, the optical properties and thermal conductivity of graphene could be also manipulated to conduct stimuli to cells. Particularly, it is possible to apply pulse laser stimulation to accelerate the neuronal differentiation of NSCs cultured on rGO films, leading to a highly interconnected network of neurons radially rearranged relatively to the laser spot.^239^ The superior behavior of cells cultured on rGO substrates comparatively to GO films was associated with the enhanced capacity of the 2D reduced platform to transfer the electrical and thermal charges toward its surface.

### Design Considerations for Scaffolding the Injured Spinal Cord

To restore the anatomical structure and physiological function of the injured spinal cord, TE scaffolds combine biomaterials, biomolecules, cells, and microfabrication techniques with the purpose of mimicking one or multiple aspects of the native ECM. It is the appropriate selection of these four factors that directs the physicochemical properties of the conceptualized scaffold toward its specific goals, including neuroprotective and/or neuroregenerative purposes. Generally, the process of designing a TE scaffold targeting SCI should take into account the following specific criterions: (1) 3D architecture; (2) biocompatibility; (3) biodegradability; (4) electrical conductivity; (5) functionalization; (6) mechanical performance; and (7) technical and economical requirements.

#### 3D Architecture

Engineered 3D architectures markedly influence cell behavior not only by conditioning the geometry of the seeded cells but also by expanding their interactivity with the surrounding microenvironment (e.g., cell–cell and cell–material interactions). Ultimately, these constructs should accurately simulate important aspects of the ongoing “dynamic reciprocity”^240^ established between cells and their natural ECM *in vivo*, including a 3D spatial organization and a set of physicochemical stimuli that induce signaling patterns resembling functional living tissues.^241^ Indeed, neural cells are capable of adapting their response to 3D architectures, showing distinctive morphological and functional features relative to 2D platforms such as (1) a random and uniform distribution of neuronal circuits through the 3D network; (2) a larger clustering coefficient combined with longer links; and (3) an enhanced neuronal calcium activity, including the formation of spontaneous synapses.^242^

The biomimetic hierarchy provided by 3D architectures critically depends on allocating topographic cues (e.g., channels, fibers, grooves, pores) across the internal structure of the scaffolds, delivering preferential routes to encourage the elongation and interconnectivity of neurites, as well as providing cavities to group neural cells into appropriate spaces. The design of the scaffold architecture should take into account both the well-defined anatomical features of the spinal cord and the variable size and shape of neural cells and blood vessels populating it. Relative to neurons in the CNS, their size ranges from 10 to 50 μm, with neurites between 0.2 and 20 μm in diameter. In terms of length, dendrites can extend up to 500 μm, while axons are able to cover distances reaching from several centimeters to a meter.^243^ The number of glial cells in the spinal cord is considerably higher compared to that of resident neurons, presenting a ratio of 12:1 in the gray matter of T8–T11 segments.^244^ Although the relative distribution of the glia cell populations differs along the spine, there is a general predominance of astrocytes (40%) and oligodendrocytes (40%), compared to microglia (20%). Astrocytes commonly present a cell body of 10 μm, with a variable number of processes with an extension between 100 and 200 μm.^245^ Regarding oligodendrocytes, they have cell bodies ranging from 6 to 8 μm that generate from 20 up to 60 myelinating processes with intermodal lengths between 20 and 200 μm.^246,247^ Microglia are smaller cells with cell bodies in the range of 3–6 μm.^248^ Besides the size of cell populations in the spinal cord, the design of the scaffold architecture should also consider the spinal vasculature, which presents arteries and veins with diameters ranging from 0.06 mm to 1 mm and from 0.4 mm to 2 mm, respectively.^249^

#### Biocompatibility

Biocompatibility is a key property for any TE scaffold. It ensures survival and functionality of the implant and its components in the host, while minimizing immune responses, toxicity-mediated cell death, and oxidative stress.^250^ Biocompatibility assessment must ponder a delicate equilibrium between the potential response triggered by the implantable scaffold and the pre-existing reactivity of the native tissue. This acquires great relevance in the particularly aggressive inflammatory response that follows SCI, which might mitigate the therapeutic effect of the scaffold.^221^ Among other pivotal actions, it is important to guarantee an appropriate surface chemistry of the selected biomaterial(s) in order to prevent any harmful effects to the cell populations resident in the spinal cord (e.g., neural, vascular, and immune), as well as to avoid collateral toxicity related to degradation subproducts. For this reason, many TE scaffolds are included in their composition natural polymers and/or FDA approved synthetic biomaterials (e.g., PCL, PLGA), capable of promoting long-term and predictable biocompatibility profiles.^251,252^

#### Biodegradability

The biodegradation rate of a scaffold should ideally match the progression of tissue regrowth, meaning that its gradual disassembly into fragments for posterior elimination should be neither too fast nor too slow in order to guarantee unrestricted cell migration, neurite outgrowth, and ingrowth of supportive tissue (e.g., ECM deposition, vascularization). There are several types of degradation processes, their activation being dependent on the chemical composition of the scaffold composing biomaterials. For example, a scaffold composed exclusively of spinal cord ECM components could instigate a rapid degradation *in vivo* (8 weeks) due to macrophage activity. Conversely, a PLGA-based scaffold (such as the “Neuro-Spinal scaffold” under clinical investigation) preferentially degrades via hydrolysis, and it is successfully reabsorbed after 12 weeks postimplantation.^253,254^ Generally, biodegradability is an optional requirement of the TE scaffold, but it is highly convenient since it annuls the need of a second injury for removal of the implant. It is important to notice that biodegradability and biocompatibility properties are closely interrelated since the progressive dissociation of the biomaterials must follow nontoxic elimination routes for organs such as the liver and the kidneys.

#### Electrical Conductivity

Although electrical conductivity is still an optional requisite for TE scaffolds targeting SCI, there is a growing interest in exploring the application of conductive biomaterials (e.g., carbon-based nanomaterials, conductive polymers, and metallic particle-based materials) in strategies that combine tissue regeneration and neuromodulation.^255^ Indeed, conductive scaffolds are capable of influencing neural cell activities, including axonal growth, differentiation, migration, and proliferation, both *in vitro* and *in vivo*, by means of electrical stimulation.^256,257^ Even in the absence of external stimuli, biomaterials with superior electrical properties can successfully boost neuronal activity. For example, as reported by Ballerini and co-workers,^258^ a MWCNTs-based film augmented the number and length of neurites arising from a spinal cord explant and significantly enhanced the synaptic activity of the neuronal network. The same group used MWCNTs to build a 3D scaffold suitable for promoting axonal growth, multisynaptic pathways, and ECM deposition toward a successful entanglement of two neurite networks outgrowing from distinct segregated spinal cord explants, leading to their functional reconnection *in vitro*.^259^ More recently, these scaffolds were successfully implanted in a SCI unilateral L1–L2 hemisection model in rats. Major findings included important advantages compared to PEG cylindrical grafts such as mechanical stability and attenuated local tissue reactivity, as well as a superior ability to support angiogenesis, boost axonal regeneration and outgrowth across the lesion area, and enhance a consistent locomotor recovery during 4 months postinjury.^260^ In detail, the improvements in motor function were quantified with a score of approximately 18 according to the BBB locomotor scale.

#### Functionalization

Optimizing the performance of TE scaffolds can be also accomplished by introducing features responsible for adding desirable functionalities. One of the most common functionalization procedures involves the attachment of ECM components (e.g., fibronectin, laminin) onto the surface of the scaffold with the purpose of improving neural cell adhesion, proliferation, and differentiation.^261,262^ Other bioactive components, including peptides like PLL and neurotrophic factors (e.g., brain-derived neurotrophic factor, interferon-γ (IFNγ), platelet-derived growth factor (PDGF)-AA), are also suitable for creating biochemical coatings to manipulate neural cells responses and support tissue development.^263,264^ Complementarily, the efficient dispersion of nanomaterials into polymer matrixes is a feasible alternative to reinforce biochemical, electrical, and mechanical features of the scaffold toward neural repair.^265,266^ For example, the presence of manganese dioxide nanoparticles in a PPFLMLLKGSTR peptide-modified HA hydrogel was crucial for moderating the SCI oxidative microenvironment *in vivo* and, subsequently, boosting the neuroregenerative impact of transplanted MSCs.^267^

#### Mechanical Performance

CNS tissues are soft tissues of the body, their structure and function being directly influenced by the maintenance of a favored biomechanical environment. Specifically, the human spinal cord presents a mechanical anisotropy related to the inherent characteristics of each anatomical region, including variations in diameter and shape.^268^ Thus, ideally, the engineered scaffold should precisely match the mechanical characteristics of the concrete spinal cord area to be replaced. In this context, Karimi *et al.*(269) have analyzed the mechanical performance of the human cervical spinal cord under unconfined compressive loading at a low strain rate (5 mm/min), reporting values of 40 ± 7 kPa and 62 ± 5 kPa for the elastic modulus and maximum/failure stress, correspondingly. Additionally, confined compression tests of *ex situ* porcine spinal cord samples revealed a superior aggregate modulus and peak stress for the gray matter (205 ± 13 kPa and 126 ± 4 kPa) relatively to the white matter (103 ± 13 kPa and 80 ± 4 kPa), respectively.^270^ In agreement with these data, atomic force microscopy (AFM)-based indentation experiments examining rat spinal cord sections also concluded that the gray matter was stiffer than the white matter (Young moduli of 275 ± 99 Pa and 97 ± 69 Pa, respectively).^271^ Although the mechanical compliance of the scaffold should target a biomimetic range, it must not compromise its functional and structural integrity, meaning that the scaffold should simultaneously guarantee an appropriate fixation *in vivo* as well as enough flexibility to adjust to the injured area without mechanically damaging healthy tissues (e.g., friction forces). Moreover, the scaffold must present a certain degree of swelling capacity to ensure permeabilization of cells, molecules, and gases throughout its entire 3D architecture.^272^

It is important to notice that the modulation of the mechanical properties of the scaffold is an attractive strategy for tailoring important cellular processes. For example, neuronal cells enhance their performance (e.g., growth, viability, neurite elongation) when seeded onto soft substrates, even being capable of responding to small variations of stiffness on the magnitude of 70 Pa.^273^ Likewise, the differentiation of both MSCs and NSCs into neurons proved to be more pronounced onto scaffolds with softer mechanical responses (1 kPa and 0.1–0.5 kPa, respectively) compared to stiffer structures (10 kPa and 1–10 kPa, correspondingly), where the seeded stem cells showed a preferential differentiation into glia.^274,275^

#### Technical and Economical Requirements

Similar to any TE application, scaffolds for SCI must present a discerning balance of important specifications regarding fabrication costs, implantability, processability, and reproducibility. Thus, the selection of the microfabrication technique(s) gains capital importance because it should, ideally, guarantee accurate control of the desired 3D architecture in a cost- and time-efficient manner. Furthermore, the technology involved in the manufacturing process must consider the processability features of the biomaterials, the suitable reproduction of both design and function of the scaffold, the availability and well-conditioned manipulation/preservation of sensitive therapeutic agents such as biomolecules and cells, and the urgency associated with the narrow and rigorous therapeutic time window following SCI. Overall, each biofabrication approach (e.g., 3D printing, electrospinning, hydrogel synthesis) offers singular points of contact among research, industrial and clinical areas, presenting, subsequently, specific advantages and disadvantages associated with biomimicry, capital/operation costs, and implantability.^276,277^ For instance, the functionality of a biomimetic scaffold could be seriously compromised if the surgeon has to substantially cut or reshape the construct before implantation with the purpose of matching the irregular geometry of the injured site. Theoretically, this particular issue might be addressed either by adapting the design of the scaffold to each lesion area, therefore augmenting the time and cost of production or by replacing the well-defined biomimetic features with an adaptable architecture suitable to adjust the size and shape *in situ*.

From a commercial-scale perspective, it is noteworthy to mention that the produced scaffolds must experience rigorous quality control before any clinical usage, including the accuracy of the final constructs (e.g., geometry, dimensions, chemical composition) and their long-term stability (e.g., “off-the-shelf availability” and storage conditions).^277^ The success of such execution is also intrinsically dependent on implementing meticulous quality assurance, marketing, and financial management policies.^278^

### Proof-of-Concept in 3D Systems

**Figure 8:**
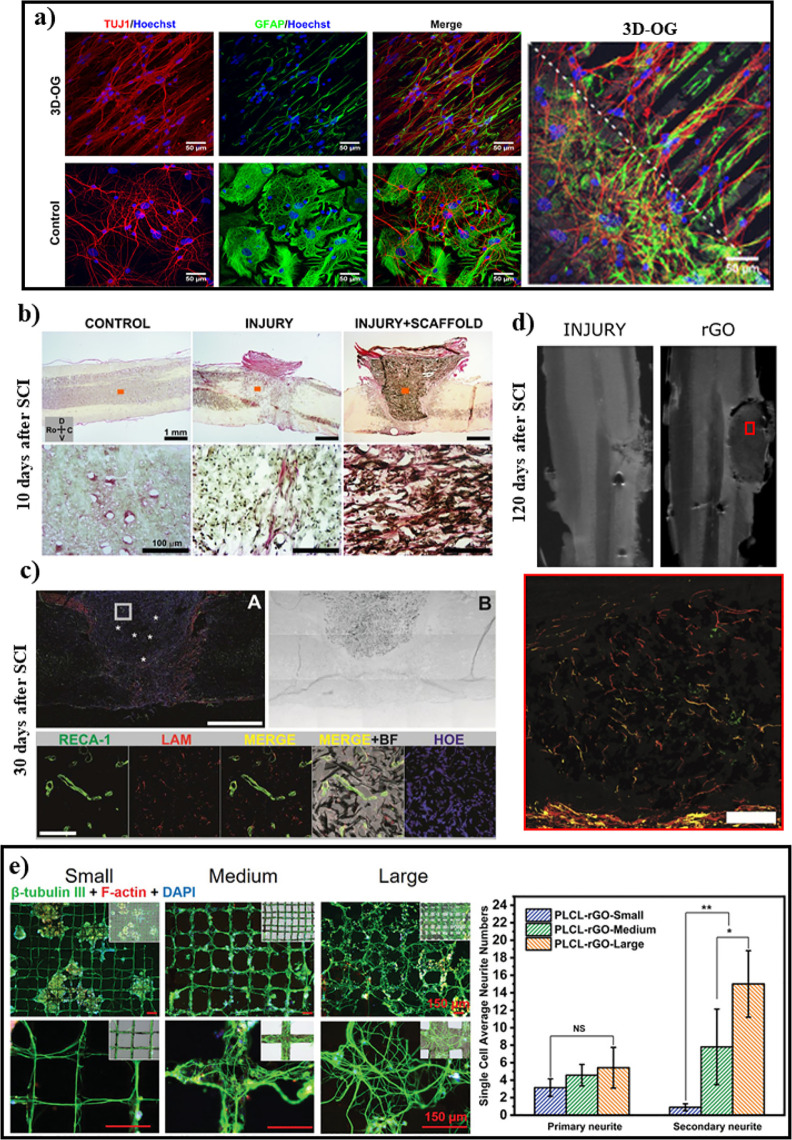
3D graphene-based scaffolds targeting TSCI. (a) Primary cortical cell growth and maturation after 8–10 days *in vitro* in 3D ordered graphene scaffolds (3D-OG) and 2D glass coverslip controls. Neurons were stained with β-III tubulin (TUJ1, red), astrocytes with GFAP (green), and cell nuclei with Hoechst (blue). On the right, it is possible to observe the geometric modulation of neuronal growth *in vitro*. The image acquired in reflection mode illustrating the 3D-OG structure was merged for proper visualization of the substrate. The part under the white line was the larger graphene skeleton supporting the whole structure, and the part above the line was the ordered graphene skeletons. Scale bars = 50 μm. Adapted with permission from ref (282). Copyright 2020 American Chemical Society. (b) 3D rGO porous scaffolds implanted at the C6 right rat hemicord at 10 days postinjury. Histological examination of the spinal cord tissue by conventional hematoxylin-van Giesson staining. Images at the bottom represent zoom-in details of areas marked with orange squares in top images. Spinal cords are oriented in all cases as indicated by the set of arrows: C–Caudal, D—Dorsal, Ro—Rostral, and V–Ventral. Scale bars = 1 mm (top) and 100 μm (bottom). Reproduced with permission from ref (283). Copyright 2015 Wiley-VCH. (c) Angiogenesis inside a 3D rGO porous scaffold at 30 days postinjury in a hemisection SCI model in rats. (A) Low-magnification view of the spinal tissue (top), blood vessels inside the scaffold structure marked with white asterisks. Zoom-in images (bottom) corresponding to the area indicated by the gray square in the top image. Mature blood vessels were detected by labeling of RECA-1 (green) and laminin from basal membranes (LAM, red). Cell nuclei were labeled with Hoechst (HOE). (B) The bright field (BF) image enables the visualization of the scaffold. Scale bars: 1 mm (top) and 100 μm (bottom). Reproduced with permission from ref (285). Copyright 2016 Elsevier. (d) Performance of a 3D rGO porous scaffold at 120 days postinjury in a hemisection SCI model in rats. MRI features of the rGO implants corresponding to coronal scans of hemisected rats without and with the rGO scaffold (top) and a representative confocal laser scanning microscopy image highlighting the impact of the scaffold on boosting neurites growth inside the scaffold (bottom). Labeling for β-III tubulin (red) and antipan-neuronal neurofilament (green) marked the cytoskeleton microtubules and neurofilaments of neuronal cells, respectively. Scale bar = 150 μm. Adapted with permission from ref (271). Copyright 2019 Elsevier. (e) PC-12 cells cultured on rGO microfibers with different diameters under electric stimulation after 14 days *in vitro*. Representative immunofluorescence images of the cells stained with β-III tubulin (green), F-actin (red), and DAPI (blue) at low (top) and high (bottom) magnification (left). Inset: Merged bright-field image. Scale bars: 150 μm. Quantitative analysis of average neurite numbers (right). Adapted with permission from ref (302). Copyright 2020 Wiley-VCH.

Different studies have highlighted the favorable response of 3D graphene-based porous scaffolds to modulate the behavior of CNS cellular components. For example, cultured hippocampal neuronal and glia cells effectively colonized the pores of a 3D graphene construct *in vitro*, leading to the recreation of complex neural networks with a wide range of active synaptic connections in which the high rate of spontaneous bursts was associated with the interference of graphene with the maturation of GABAergic inhibition.^279^ Effectively, neuronal circuit dynamics was influenced by the establishment of 3D neuron–graphene interactions responsible not only for increasing the synchronization of the synaptic activity, including transitions between highly and moderately synchronized regimes, but also for promoting the interconnectivity among active neurons by inciting neurite elongation to cover long distances through and cross the pores.^280^ 3D porous structures fabricated by advanced CVD modalities could even modulate neural cell behavior *in vitro* according to rigorous geometrical disposition of graphene building blocks, including their skeleton dimension and orientation angle, as well as pore size. In fact, smaller graphene skeleton widths (10 and 50 μm of spacing) boosted neuronal differentiation of NSCs and supported a superior number of astrocytic processes relatively to higher skeletons widths (20 and 50 μm).^281^ Similarly, Xiao *et al.*(282) optimized the dimensions of a 3D ordered graphene scaffold (pore size and skeleton width of 20 μm and an orientation angle of 90°) with the purpose of recreating an *in vivo*-like neural microenvironment from cultured primary cortical cells. Results showed that the orientation of the graphene skeletons favored the elongation of both axons and dendrites, as the nanosized topographical cues (ripples and wrinkles) were responsible for inducing branched astrocyte morphologies (Figure 8a). After 8 days *in vitro*, highly active neuronal cells proved to trigger signaling pathways over long distances along the ordered graphene skeletons.

It seems undoubtable that advances in the exploration of 3D GBMs for neural TE continues to grow at a rapid pace. Table S3 summarizes the most significant progress in the design and performance of 3D graphene-based scaffolds focused on spinal cord TE applications. In 2015, Serrano and co-workers^283^ pioneered the implantation of a graphene-based scaffold in an experimental model of SCI in rats (C6 right hemisection). From this first study addressing the subacute phase of the SCI pathophysiology (10 days of implantation), it was concluded that the tested 3D rGO porous scaffold was nontoxic and able to suitably integrate into the host environment (Figure 8b), promoting the infiltration of connective tissue (i.e., collagen) together with the migration of cells, which were predominantly non-neuronal, within the construct. The development of this rGO scaffold relied on the application of the ice segregation-induced self-assembly technique, during which a GO aqueous solution was unidirectionally frozen and then lyophilized in order to engineer a 3D porously channeled network that was further cross-linked with hexamethylene diisocyanate vapors and thermally reduced for optimizing its final biochemical, mechanical, and structural features.^284^ Indeed, as it was proven by preliminary *in vitro* tests, the final 3D rGO porous system showed an indisputable capacity for efficiently interacting with neural cells proven by the enhanced response of embryonic neural progenitor cells (ENPCs) (e.g., formation of highly viable neural networks composed by a majority of neurons accompanied by glial cells).

These promising findings further encouraged this group to extend the period of implantation to 30 days, with the purpose of evaluating the response of the injured microenvironment at an early chronic stage.^285^ In this case, an equivalent rGO scaffold with random porosity was decisive to prevent the formation of cavities in the lesion site and, subsequently, to guarantee the stabilization and selling of the SCI. The maintenance of its 3D porous architecture *in vivo* was capable of sustaining variations in the infiltrated cell populations relatively to the subacute phase, considering the overall decrease of vimentin positive cells notwithstanding the augmentation of M2/M1 macrophages ratio. Additionally, the homogeneous rostro-cauda distribution of RECA-1 and laminin confirmed the formation of blood vessels at the interface and interior of the rGO scaffold (Figure 8c), leading to the establishment of a pro-regenerative microenvironment supporting the outgrowth of some axons through the porous system. Finally, there were no signs of systemic toxicity induced by the rGO material since the histological evaluation of the major organs (i.e., liver, kidneys, and spleen) did not revealed significant alterations compared to control rats. The regrowth of neurites within the rGO scaffold was also confirmed *in vivo* after 4 months of implantation in hemisected rats (Figure 8d).^271^ These attempts of spinal reconnection from the projected myelinated excitatory axons were observed near the formed blood vessels, indicating a plausible direct correlation between neurite regeneration and angiogenesis in the chronic stage of the disease. Furthermore, this freeze-dried rGO system was capable of integrating the host tissue without either interfering with the spontaneous behavioral features of the tested rats or inducing systemic toxicity, and even showing incipient signs of scaffold disassembly and eventual rGO biodegradation. It is also important to notice that a pioneer MRI analysis was conducted to contextualize and estimate the morphological changes at the injury epicenter and any potential radiological evidence of damage at the perilesional areas provoked by the presence of the scaffold. Results shown that, although the rGO porous system contributed to a significant enlargement of the lesion epicenter, it was responsible for substantially decreasing the volume and number of perilesional damage areas. The reported AFM findings pointed out that this enhanced interface with the native neural tissue was associated with the softness of the scaffold (Young modulus = 1.3 ± 1 kPa), which successfully guaranteed biomimetic mechanical compliance *in vivo* and prevented any significant damage to the surrounding spinal cord microenvironment.

It is important to notice that these *in vivo* investigations with rGO porous scaffolds at the injured spinal cord were performed in the absence of any functionalization, cell/drug delivery, or external stimuli.^271,283,285^ Thus, there are a few possible routes that could be explored in a near future scenario to, eventually, boost these already promising pro-regenerative results. For instance, the combination of GBMs with natural polymers such as alginate, collagen, and chitosan via either chemical or physical cross-linking could promote enhanced neural cell responses while enabling the manipulation of important features of the resultant 3D scaffolds such as biocompatibility/biodegradability profiles, electrical properties, mechanical compliance, structural stability, and porosity.^286−288^ For instance, a recently reported 3D graphene-collagen cryogel moderated the presence of inflammatory cytokines when subjected to an inflammatory model *in vitro*, as well as induced a positive M2/M1 macrophage ratio.^289^ Complementarily, this scaffold improved the neuronal differentiation of cultured bone marrow MSCs via electrical stimulation and, importantly, established a bioactive interfacing with 3D organotypic spinal explants responsible for supporting the infiltration and proliferation of neural cells across the graphene-collagen porous network. In a different study, the presence of GO in the chemical composition of a 3D chitosan porous system slowed down its in vivo degradation, as evidenced by a lower increase of the mean pore diameter (from 37 to 79 μm) relatively to pure chitosan scaffolds (from 58 to 110 μm) after 10 weeks postimplantation.^290^ Such pores supported pronounced angiogenesis and tissue ingrowth in the chitosan-GO structure, likely related to the boosted locomotor performance found (BBB score = 8–9).

Additionally, the topographical cues of graphene-based scaffolds could be expanded with the integration of nanofibrous components, such as bacterial cellulose, into the graphene porous skeleton, leading to a 3D scaffold capable of improving the density, functionality, and viability of the resident neural cultures by guiding the elongated neurites through these complementary topologies.^291^ Following this trend, a recent approach described that electrospun nanofibers could be easily mixed with rGO nanosheets to generate a hydrogel-like structure suitable for providing a 3D combinatorial fibrous-porous architecture after lyophilization.^292^ In fact, the walls of the pores of the final scaffold presented a nanofibrous coating responsible not only for influencing the mechanical and structural features of the construct but also for enhancing ENPCs adhesion and differentiation. It was suggested that the presence of the nanofibers onto the surface of the rGO sheets was a decisive factor to guide and expand the elongated neurites and, subsequently, to form a highly viable and interconnected 3D neural network *in vitro*.

There is an undeniable attractiveness to consider bioactive and electrical enhanced graphene-based fibrous scaffolds as top candidates for directly guiding the outgrowth of regenerated neurites in the context of SCI. A promising example was reported by González-Mayorga *et al.*,^293^ who developed microfibers exclusively made of rGO and evaluated their performance *in vivo* during the subacute stage of the SCI pathophysiology. Prior to implantation, the physiochemical characteristics (e.g., diameter, electrical conductivity, and surface roughness) of these rGO structures were optimized by adapting the temperature of the one-step dimensionally confined hydrothermal methodology. The resulting microfibers were able to efficiently immobilize biomolecules like PLL and N-cadherin onto their surfaces and support highly viable neural networks composed of differentiated neurons and glia *in vitro*. After 10 days of implantation in the hemisected spinal of a rat model, rGO microfibers (without any biological coating) proved to be able to suitably interface the SCI, considering the notorious colonization by non-neuronal cells, including glia, and the absence of systemic toxic effects. To better understand the potential of these rGO microfibers as neuroregenerative agents, the response of macrophages was evaluated *in vitro*, revealing high cell viability, as well as a reduction of their proliferative capacity, and a time-dependent polarization into M1 (after 24 h) and M2 (48 h) phenotypes.^294^ Moreover, Domínguez-Bajo *et al.*(295) conducted a decisive study to validate this scaffolding approach by implanting these rGO microfibers into the rat cervical injured spinal cord for 4 months. Results shown that the presence of the microfibers supported the existence of viable neurons and blood vessels at the epicenter of the SCI without triggering any negative outcomes regarding inflammation, functional recovery, and systemic toxicity.

Despite these promising *in vitro* and *in vivo* results, the implementation of the reported rGO microfibers^293,295^ presupposed their manual integration within a gelatin hydrogel (approximately 20 microfibers per hydrogel) to guarantee an appropriate dimensionality and rostro-caudal orientation as well as to enable suitable manipulation during surgery. The supportive role of the gelatin hydrogel is maintained for roughly 2 months due to the biodegradation profile of this natural polymer *in vivo*. Taking this into account, it might be possible to optimize this scaffolding concept by using fabrication methodologies such as 3D printing capable for accurately designing the microfibrous system (e.g., dimensions, orientation, and 3D rearrangement) in a feasible and reproducible manner. Considering the diameter of the reported rGO microfibers (>100 μm),^293,295^ there are highly functional inks composed entirely of GBMs that have already proved to efficaciously print several microstructures within a similar scale, including both 3D graphene porous and microfibrous systems.^296−298^ Nevertheless, both the application of these graphene inks and the development of 3D neural TE scaffolds from printable graphene-based composites are still in their infancy.^299^ Some progress in this matter includes the use of GBM-polyurethane printed scaffolds to mediate NSCs differentiation *in vitro*(300) and graphene-PCL printed structures to stimulate regeneration of injured peripheral nerves *in vivo*.^301^ To avoid the drawbacks usually associated with graphene-based inks (e.g., dispersibility and discontinuity of the nanomaterials), microfibrous systems could be also coated with GBMs after printing. For example, Wang *et al.*(302) have successfully used near-field electrostatic printing to produce poly(l-lactic acid-*co*-caprolactone) microfibrous systems with a wide range of designs. Their bulk properties (e.g., conductivity, surface chemistry, and roughness) were then enhanced by a layer-by-layer GO coating followed by *in situ* reduction. The final scaffold supported an interconnected and functional neuronal network *in vitro*, whose characteristics were efficiently modulated by modifying the diameter of the microfibers (i.e., 17, 72, and 150 μm), smaller fibers being responsible for inducing larger neurites and delivering electrical stimuli for further enhancing oriented neurites outgrowth and branching (Figure 8e).

Similarly, electrospun polymeric micro-/nanofibers can be successfully enhanced by the incorporation of GBMs into their chemical composition or, alternatively, after immobilization of these nanomaterials onto their surfaces. The resultant interaction with neural cells has proved to be easily adjustable according to the content of GBMs and/or electrical stimulation patterns, showing auspicious results regarding (1) the acceleration of neurite outgrowth; (2) the improvement of cell adhesion, metabolism and proliferation; and (3) the unlocking of more efficient differentiation pathways toward specialized cellular lineages like neurons and oligodendrocytes.^303−306^ Even though the majority of the reported graphene-based electrospun scaffolds for neural TE has been specifically designed to enhance PNS regeneration, it was recently highlighted that aligned silk fibroin-graphene composite electrospun fibers supported the growth of viable spinal cord neurons *in vitro* and, interestingly, that the presence of graphene boosted the expression of Netrin-1, a protein associated with axonal outgrowth.^307^ GBMs also proved to be capable of efficiently arbitrating the interactions of PCL–gelatin composite electrospun scaffolds with ENPCs, both being the chemical composition and size of the nanomaterials major influencers on promoting favorable cell responses.^292^ Indeed, compared to PCL-gelatin-GO and PCL-gelatin-rGO synthesized with small rGO flakes, the PCL-gelatin-rGO electrospun system was significantly more competent to adsorb PLL and, subsequently, to induce appropriate morphology and high viability in ENPCs. Relatively to *in vivo* studies, Pan *et al.*(308) successfully covered the hemisected area of a rat SCI model with a PLGA-GO electrospun mesh for the local delivery of both insulin-like growth factor 1 and brain-derived neurotrophic factor, promoting functional recovery (BBB score = 16–18) and reduction of the lesion cavity after 4 weeks of implantation. In another report, Zhou *et al.*(309) modified the surface of PCL electrospun microfibers with sequential layers of heparin/graphene and PLL with the purpose of attenuating astrocyte and microglia activation after brain implantation in a rat model. Results confirmed that graphene functionalization was responsible for boosting the infiltration of astrocytes into the scaffold without augmenting their reactivity levels, thus preventing glial scarring during 7 weeks *in vivo*.

It is important to notice that the ability of GBMs to integrate SCI drug delivery approaches might hold a particular interest for neuroprotective strategies as well. For instance, a hydrogel composed by GO and 4arm-PEG-diacerein could be successfully injected into the SCI with the purpose of releasing diacerein in a controlled manner for modulating the endogenous inflammatory response.^310^ In addition to the reduction of astrocyte reactivity, inflammation, and lesion area, a significant neuronal/axonal regeneration was evidenced, as well as locomotion recovery (BBB score = 13) of tested rats after 28 days postinjection. In a different approach, Chiang *et al.*(311) used a layer-by-layer assembly technique to functionalize porous PLGA microcapsules with rGO and silk-PLL in order to improve their conductivity, cytocompatibility, and mechanical properties toward accurate release of NT3 into the injured spinal cord. This advanced system was further integrated into a hierarchical hybrid gelatin methacrylate-microcapsule hydrogel with an optimized triangular shape that allowed not only a suitable implantation but also a more efficient spatiotemporal delivery of the loaded biomolecules. The enhanced migration and neuronal differentiation of NSCs *in vivo* was likely responsible for the augmentation of the locomotor performance of injured rats after 35 days of implantation (BBB score = 16).

Considering all these encouraging results, 3D graphene-based scaffolds could assume the role of central players in SCI approaches in a near future scenario. Indeed, constructs synthesized from GBMs, particularly rGO, have promoted exciting neuroregenerative and neuroprotective effects both *in vitro* and *in vivo* such as angiogenesis, axonal growth, reduction of the injury scar, and moderation of inflammatory responses. Since such outcomes were mainly accomplished by simple scaffolding approaches with little or no emphasis on additional therapeutic agents (e.g., cells, drugs, bioactive molecules, chemical functionalizations), many potential paths remained to be explored in order to modulate the highly complex SCI microenvironment. Furthermore, the current investment on fundamental studies targeting neural cell–GBM interactions, as well as the development of more biomimetic 3D architectures via advanced technologies, could boost the modulation of cell responses via accurate biochemical and biophysical gradients purposely fabricated according to each lesion. However, it is important to note that the enthusiasm surrounding the control of neural systems with GBMs should be skeptically regulated not only by the results coming from longer times of study but also by the gradual understanding of the physicochemical properties of GBMs, which could anticipate long-term toxic effects triggered via multiple biodegradation routes.

## Final Remarks and Future Perspectives

Currently, there are no available medical treatments that completely reverse the tragic consequences of SCI. Since diagnosis, the majority of SCI patients suffer a multitude of devastating physiological, emotional, and social consequences that modern medicine is only mitigating via surgical procedures, pharmacological therapies, and rehabilitation protocols, only resulting in palliative outcomes. Importantly, ongoing clinical trials show the potential to revert such adverse panorama in a near scenario, including the administration of neuroprotective drugs for attenuating the SCI inflammatory cascade, as well as the application of neuromodulatory stimulation and cell-based therapies to boost/awaken endogenous neuroregenerative mechanisms. However, it is important to note that the current impact of clinical trials targeting SCI, although appreciable, might be optimized via two complementary routes: (1) a deep effort on the performance of more complete and updated epidemiologic studies to assist the adaptation of common international standards for guaranteeing comparable results regarding classification, follow-up, and rehabilitation of SCI patients (of interest for better matching and selection of candidates for clinical trials and the contextualization of their evolution) and (2) a robust implementation of adaptive designs for SCI clinical trials^312^ to diminish inherent limitations of conventional randomized controlled trials such as low incidence, high heterogeneity, dubious methods to undoubtedly prove treatment efficiency, and elevated costs per candidate.

Considering the absence of ongoing clinical trials addressing the efficacy/safety of GBMs in SCI therapeutics, as well as the average time and cost that a drug (10 years and ∼1 billion dollars) or a medical device (7 years and millions of dollars) takes to be approved by the FDA^313^ (or respective counterparts such as the European Medicines Agency), it becomes clear that interfacing the injured spinal cord with graphene-based platforms is still far from becoming a reality for patients. This is particularly evident considering the lack of literature describing the adaptation of promising graphene-based technologies (e.g., GQDs, GO-based nanocarriers, and graphene-based electrodes) to the SCI microenvironment, despite promising interactions with neural cells and tissues. Based on this, future studies should challenge, for instance, (1) the ability of GQDs to maintain accurate and long-term labeling of transplanted neural stem cells within the SCI area; (2) the stability and precision of GO-based nanocarriers to deliver therapeutics into the injured spinal cord according to distinct degrees of functionalization and time windows of administration; and (3) the performance of graphene-electrodes not only when interfacing the mechanical dynamics of the spinal cord but also when countering the physiological variations provoked by the scar tissue at the injured spinal cord.

When specifically referring to TE approaches, some of the most auspicious hypothesized graphene-based strategies to recover the injured spinal cord have gravitated around the recovery of lost motor functionalities and the eventual elucidation of the molecular/cellular mechanisms that could help mitigate SCI pathophysiology. Particularly, the development of 3D graphene-based scaffolds is giving rise to various neuroregenerative approaches by combining advanced microfabrication techniques with biomaterial designs capable of influencing the injured neural microenvironment. Such constructs have been proven to be capable of properly sustaining viable and functional neural networks *in vitro*, further expanding their performance *in vivo* by instigating angiogenesis, axonal growth, and local delivery of biomolecules, among others. It is then plausible that these results boost a more significant progress in the next few years based on the still enormous set of possible design approaches and functionalization strategies to be explored with the potential to upgrade the interface between graphene-based scaffolds and the injured spinal cord.

As the scaffolding design guidelines evolve together with the functionality profile of the GBMs/biomaterials/cells interactions, it is further necessary to uniformize key features of both *in vitro* and *in vivo* experiments (e.g., cell culture protocols, functionalization routes, injury models) to facilitate clinical translation. It is worth noting that experimental successes in preclinical animal models may not directly correlate to such in humans mainly because of the physiological disparities among them and, importantly, the higher level of complexity of human SCI (e.g., arrangement of the endogenous neuronal circuits, immune responses, and intrinsic neural plasticity). Indeed, it is challenging to project graphene-based scaffolding approaches for regenerating the injured spinal cord due to (1) the conflicting combination of advantages and disadvantages of each 3D architecture (e.g., fibrous networks, injectable hydrogels, porous systems) regarding biomimetics, functionality, reproducibility, and implantation; and (2) the many challenges that arise from their short- and long-term biocompatibility profiles that must be carefully addressed and disclosed. Thus, there is an urgent need to establish consistent relationships among the chemical composition, adopted microfabrication techniques, and resultant biological responses of these 3D scaffolds when interfacing the injured spinal tissue. Soon, it would be possible to feed computational models to identify the scaffold parameters that best fit the demands of each SCI patient according to the principles of personalized medicine.

All things considered, it is highly plausible that GBMs start playing, sooner than expected, an important role in supporting multifunctional biomedical platforms capable of articulating complementary SCI therapeutic approaches such as cell imaging, biomolecules/drug delivery, electrophysiological recording, electrical/optical stimulation, and, importantly, neuroregeneration from enhanced neural cell–material interactions. The auspicious performance of GBMs, together with the growing enthusiasm of the scientific community working with these materials, might well shorten the long road ahead toward clinical translation.

## Supporting Information Available

Chapter describing the epidemiology of SCI (Chapter S1); a table compiling the most significant clinical trials targeting SCI (Table S1); a table summarizing the interactions between GBMs and neural cells (Table S2); and a table with promising strategies regarding the use of 3D graphene-based scaffolds targeting SCI (Table S3) (PDF)

## Vocabulary

Axon: The longest cytoplasmatic projection of the neuronal soma. It is responsible for propagating action potentials from the trigger zone to the presynaptic terminals, where the information is transmitted to neighboring neurons and peripheral effectors (e.g., motor unit)
Graphene-based materials: Class of 2D carbon nanomaterials, including pristine graphene, graphene oxide, and reduced graphene oxide, that share a basic chemical structure composed of a honeycomb lattice of carbon atoms with sp^2^ hybridization arranged in one atom-thick planar sheets. They all present a variable set of properties depending on their chemical and structural composition
Scaffold: Three-dimensional implantable system engineered to replace a diseased or damaged tissue of the body, providing structural and functional support to cells, blood vessels, and extracellular matrix components
Spinal cord injury: Damage to the spinal cord with a traumatic or nontraumatic origin responsible for the temporal or permanent loss of autonomous, motor, and/or sensory functions bellow the neurological level of injury
Stem cells: Cells with potential to differentiate toward specialized cell lineages. According to their potency, they can be classified as pluripotent, multipotent, oligopotent, and unipotent
Synapse: Signal transmission through junctions between presynaptic and postsynaptic neighboring neurons. The central nervous system predominantly utilizes chemical synapses, meaning one-way conduction of information via neurotransmitters
Tissue engineering: Interdisciplinary research field that combines principles from chemical science, clinical medicine, materials science, and nanotechnology to develop regenerative strategies for recovering damaged or nonfunctional tissues in the body. Such approaches extensively rely on the synergistically application of biomaterials, biomolecules, cells, and drugs.
