# Supplementary material for: Is Graphene Shortening the Path toward Spinal Cord Regeneration?
Source: ACS Nano. 2022 Aug 24;16(9):13430–67. doi: 10.1021/acsnano.2c04756 (PMC9776589; doi:10.1021/acsnano.2c04756)
Supplement: Supplementary file 1 — nn2c04756_si_001.pdf [file nn2c04756_si_001.pdf]

*Supporting Information*

# Is Graphene Shortening the Path toward Spinal Cord Regeneration?

*André F. Girão*, María Concepcion Serrano*, António Completo, and Paula A. A. P. Marques**

A. F. Girão, A. Completo, P. A. A. P. Marques
Centre for Mechanical Technology and Automation (TEMA), Department of Mechanical Engineering, University of Aveiro (UA), Aveiro, 3810-193, Portugal
E-mail: andrefgirao@ua.pt; paulam@ua.pt

A. F. Girão; M. C. Serrano
Instituto de Ciencia de Materiales de Madrid (ICMM), Consejo Superior de Investigaciones Científicas (CSIC), Calle Sor Juana Inés de la Cruz 3, Madrid, 28049, Spain
E-mail: mc.terradas@csic.es

# Chapter S1: Epidemiology of SCIs

A detailed epidemiologic status of SCI was reported by Halvorsen *et al.*,[1-2] who were able to analyze the profiles of SCI patients in Norway from 2012 to 2016. During this period, 69 % of patients were diagnosed with TSCI and 31 % with NTSCI, corresponding to variations between 11.4 – 15.9 and 7.7 – 10.4 patients per million population, respectively. In both categories, the majority of the patients were men (76 % for TSCI and 59 % for NTSCI), showing, nevertheless, dissimilarities in age (47 years for TSCI and 55 years for NTSCI) and in the average hospitalization time periods (120 days for TSCI and 97 days for NTSCI). The neurologic impairments were more severe for TSCI patients, whose tetraplegia / paraplegia distribution was significantly higher (48 % / 42 %) relatively to NTSCI patients (22 % / 69 %). Regarding the aetiology, this analysis concluded that TSCI was mainly due to falls (47 %), followed by sports (21 %) and traffic accidents (18 %). Similar studies corroborated that falls were the leading cause of TSCI in other European countries such as Finland (62 %), Ireland (61 %), Italy (45 %) and Spain (44 %), suggesting that population aging is a decisive factor for such prevalence relatively to other aetiologies like traffic accidents (19 % in Finland, 10 % in Ireland, 40% in Italy and 37 % in Spain).[3-6] In Japan, the most elderly society in the world, the average age of the patients was 65.9 years, being both falls at the level surface (32 %) and low-level falls (22 %) the most common causes of TSCI during 2012 and 2016.[7] A contrasting scenario is registered in the United States of America (USA), where, since 2015, traffic accidents (39 %) are provoking more TSCI cases comparatively to falls (32 %), violence (14 %) or sports (8 %). Furthermore, statistically, the most frequent SCI diagnosis in the USA is incomplete tetraplegia (47 %), followed by complete paraplegia (20 %), incomplete paraplegia (20 %) and complete tetraplegia (12 %).[8]

Although each epidemiologic study regarding SCI is associated to specific limitations (*e.g.* inclusion criteria, access to updated hospital databases and legal permissions), it is possible to assume that younger groups appear to be more susceptible to accidents associated to traffic, violence and sports; while domestic falls, malignant tumours (malignant NTSCI) and other non-traumatic mechanisms like infections (non-malignant NTSCI) are more common in the elderly population. Considering population aging, there is a growing interest in updating the epidemiological data on NTSCI, which is currently scarce. Yet, a study conducted in Switzerland stated that patients suffering from this condition presented a mortality rate 1.6 times higher comparatively to the general population. Moreover, the cause of death differed according to the origin of the disease, being neoplasms (88 %) and cardiovascular disorders (40 %) the most significant for malignant and non-malignant NTSCI, respectively.[9] The impact of NTSCI is already noticeable in Ireland, where the incidence of cases (27 per million population in 2017) was considerably higher than TSCI (13 per million population in 2016). According to this epidemiologic analysis, NTSCI was mainly provoked by degenerative disorders (49 %), neoplastic conditions (26 %) and infections (12 %), leading to a diagnosis of incomplete paraplegia for the majority of the patients (84 %).[10]

## References


(1) Halvorsen, A.; Pettersen, A. L.; Nilsen, S. M.; Halle, K. K.; Schaanning, E. E.; Rekand, T. Non-traumatic spinal cord injury in Norway 2012–2016: analysis from a national registry and comparison with traumatic spinal cord injury. *Spinal Cord* **2019,** *57*, 324-330.

(2) Halvorsen, A.; Pettersen, A. L.; Nilsen, S. M.; Halle, K. K.; Schaanning, E. E.; Rekand, T. Epidemiology of traumatic spinal cord injury in Norway in 2012–2016: a registry-based cross-sectional study. *Spinal Cord* **2019,** *57*, 331-338.

(3) Johansson, E.; Luoto, T. M.; Vainionpää, A.; Kauppila, A.-M.; Kallinen, M.; Väärälä, E.; Koskinen, E. Epidemiology of traumatic spinal cord injury in Finland. *Spinal Cord* **2020**.

(4) Franceschini, M.; Bonavita, J.; Cecconi, L.; Ferro, S.; Pagliacci, M. C.; Bellentani, M.; Cavina, A.; Biggeri, A.; De Iure, F.; Gordini, G.; Redaelli, T.; Actis, M. V.; Del Popolo, G.; Bertagnoni, G.; Avesani, R.; Falabella, V.; for the Italian, S. C. I. S. G. Traumatic spinal cord injury in Italy 20 years

later: current epidemiological trend and early predictors of rehabilitation outcome. *Spinal Cord* **2020,** *58*, 768-777.
(5) Bárbara-Bataller, E.; Méndez-Suárez, J. L.; Alemán-Sánchez, C.; Sánchez-Enríquez, J.; Sosa-Henríquez, M. Change in the profile of traumatic spinal cord injury over 15 years in Spain. *Scandinavian Journal of Trauma, Resuscitation and Emergency Medicine* **2018,** *26*, 27.
(6) Smith, É.; Fitzpatrick, P.; Lyons, F.; Morris, S.; Synnott, K. Prospective epidemiological update on traumatic spinal cord injury in Ireland. *Spinal Cord Series and Cases* **2019,** *5*, 9.
(7) Kudo, D.; Miyakoshi, N.; Hongo, M.; Kasukawa, Y.; Ishikawa, Y.; Ishikawa, N.; Shimada, Y. An epidemiological study of traumatic spinal cord injuries in the fastest aging area in Japan. *Spinal Cord* **2019,** *57*, 509-515.
(8) *National Spinal Cord Injury Statistical Center, Facts and Figures at a Glance. Birmingham, AL: University of Alabama at Birmingham, 2020.*
(9) Buzzell, A.; Chamberlain, J. D.; Eriks-Hoogland, I.; Hug, K.; Jordan, X.; Schubert, M.; Zwahlen, M.; Brinkhof, M. W. G.; for the Swi, S. C. I. s. g.; the Swiss National, C. All-cause and cause-specific mortality following non-traumatic spinal cord injury: evidence from a population-based cohort study in Switzerland. *Spinal Cord* **2020,** *58*, 157-164.
(10) Smith, É.; Fitzpatrick, P.; Lyons, F.; Morris, S.; Synnott, K. Epidemiology of non-traumatic spinal cord injury in Ireland – a prospective population-based study. *The Journal of Spinal Cord Medicine* **2020**, 1-6.

**Table S1.** Most Remarkable and Recent Clinical Trials Targeting SCI.

| Therapeutic agent | Title | Start date | Phase / Status | Major findings | ClinicalTrials.gov identifier / Country |
|---|---|---|---|---|---|
| Decompressive surgery (NP) | Prospective Study for Delay in Surgical Treatment of Traumatic Cervical Central Cord Injury in Canal Stenosis | July 2016 | N/A Recruiting | N/A | NCT02673320 France |
| Systemic hypothermia (NP) | Systemic Hypothermia in Acute Cervical Spinal Cord Injury - A Prospective, Multi-center Case Controlled Study | August 2017 | N/A Recruiting | N/A | NCT02991690 USA |
| Minocycline (antibiotic, NP) | A Pilot Study to Assess Clinical Safety and Tolerance of Minocycline and Spinal Perfusion Pressure Augmentation in Acute Spinal Cord Injury | June 2004 | Completed (August 2010) | Patients improved motor function | NCT00559494 Canada |
| Riluzole (NP) | A Multi-Center, Randomized, Placebo Controlled, Double-Blinded, Trial of Efficacy and Safety of Riluzole in Acute Spinal Cord Injury | October 2013 | Phases II/III Terminated | Preservation of neural tissue | NCT01597518 Several |

| | | | | | |
|---|---|---|---|---|---|
| Fibroblast growth factor-analog (SUN13837) (NP) | A Multicenter, Randomized, Double-Blind, Placebo-Controlled, Parallel-Group Study to Evaluate the Efficacy, Safety, and Pharmacokinetics of SUN13837 Injection in Adult Subjects With Acute Spinal Cord Injury | August 2012 | Phase II Completed | Patients improved sensory and motor functions | NCT01502631 Several |
| Nogo antibody (NG-101) (NR) | Antibodies Against Nogo-A to Enhance Plasticity, Regeneration and Functional Recovery After Spinal Cord Injury - A Multicenter International Randomized Double Blind Placebo Controlled Phase II Clinical Proof of Concept Trial | May 2019 | Phase II Recruiting | N/A | NCT03935321 Several |
| Cethrin/VX-210 (NR) | A Phase IIb/III, Double-blind, Randomized, Placebo Controlled, Multicenter Study to Assess the Efficacy and Safety of VX-210 in Subjects With Acute Traumatic Cervical Spinal Cord Injury | February 2016 | Phases II/III Terminated | Patients improved the performance of some functional tasks | NCT02669849 USA and Canada |
| Epidural SCS (NR) | Epidural Stimulation for Spinal Cord Injury | August 2017 | N/A Recruiting | Patients improved their sustained volitional movement | NCT03026816 USA |

| | | | | | |
|---|---|---|---|---|---|
| Epidural SCS (NR) | Epidural Stimulation for Spinal Cord Injury | July 2016 | N/A Active, not recruiting | Patients were able to perform complex motor functions | NCT02936453 Switzerland |
| Transcutaneous SCS (NR) | Non-Invasive Spinal Cord Stimulation and Respiratory Plasticity | May 2018 | N/A Recruiting | N/A | NCT03998527 USA |
| Transcranial direct current stimulation (NR) | SCIMS Project 1: Enhancing Corticospinal Activation for Improved Walking Function | March 2017 | N/A Completed | N/A | NCT03237234 USA |
| BCI (exoskeleton) (SMF) | Brain Computer Interface: Neuroprosthetic Control of a Motorized Exoskeleton | September 2015 | N/A Recruiting | Patients performed functional tasks and locomotion | NCT02550522 France |
| BCI (neuromuscular electrical stimulation) (SMF) | Reanimation in Tetraplegia | November 2013 | N/A Completed | Patients performed complex movements | NCT01997125 USA |
| BCI (intracortical microstimulation) (SSMF) | A Sensorimotor Microelectrode Brain-Machine Interface for Individuals With Tetraplegia | December 2013 | N/A Recruiting | Patients recognized and localized pressure-like stimulus and | NCT01894802 USA |

| | | | | | |
|---|---|---|---|---|---|
| | | | | performed complex movements | |
| Autologous human Schwann cells (NR) | The Safety of Autologous Human Schwann Cells in Subjects With Subacute Spinal Cord Injury | November 2012 | Phase I Completed | No adverse events | NCT01739023 USA |
| Autologous human Schwann cells (NR) | The Safety of Autologous Human Schwann Cells in Subjects With Chronic Spinal Cord Injury Receiving Rehabilitation | January 2015 | Phase I Completed | No adverse events nor impediments to participate in training programs | NCT02354625 USA |
| Autologous bone marrow-derived MSCs (NR) | A Phase II/III Clinical Trial to Evaluate the Safety and Efficacy of Bone Marrow-derived Mesenchymal Stem Cell Transplantation in Patients With Chronic Spinal Cord Injury | August 2012 | Phase II/III Terminated | Patients improved motor function | NCT01676441 Republic of Korea |
| Autologous human spinal cord-derived NSCs (NR) | A Phase I, Open-label, Single-site, Safety Study of Human Spinal Cord-derived Neural Stem Cell Transplantation for the Treatment of Chronic SCI | January 2013 | Phase I Recruiting | N/A | NCT01772810 USA |

| | | | | | |
|---|---|---|---|---|---|
| Neuro-Spinal scaffold (NR) | The INSPIRE Study: InVivo Study of Probable Benefit of the Neuro-Spinal Scaffold™ for Safety and Neurologic Recovery in Subjects With Complete Thoracic AIS A Spinal Cord Injury | October 2014 | N/A Active, not recruiting | Patients improved their neurological status | NCT02138110 USA |
| Neuro-Spinal scaffold (NR) | Randomized, Controlled, Single-blind Study of Probable Benefit of the Neuro-Spinal Scaffold™ for Safety and Neurologic Recovery in Subjects With Complete Thoracic AIS A Spinal Cord Injury as Compared to Standard of Care | December 2018 | N/A Recruiting | N/A | NCT03762655 USA |
| NeuroRegen scaffold + bMMCs or MSCs (NR) | Safety and Efficacy of NeuroRegen Scaffold™ With Bone Marrow Mononuclear Cells or Mesenchymal Stem Cells for Chronic Spinal Cord Injury Repair | January 2015 | Phase I Enrolling by invitation | N/A | NCT02352077 China |
| NeuroRegen scaffold + bMMCs (NR) | NeuroRegen Scaffold™ With Bone Marrow Mononuclear Cells Transplantation *vs.* Intradural Decompression and Adhesiolysis in Patients With Chronic Spinal | January 2016 | Phase I/II Enrolling by invitation | N/A | NCT02688062 China |

| | | | | | |
|---|---|---|---|---|---|
| NeuroRegen scaffold (NR) | Safety and Efficacy of Functional Neural Regeneration Collagen Scaffold Transplantation in Complete (AISA) Acute Spinal Cord Injury Patients | July 2015 | Phase I Recruiting | Patients improved sensory and motor functions | NCT02510365 China |
| NeuroRegen scaffold + MSCs or NSCs (NR) | The Efficacy and Safety of NeuroRegen Scaffold™ Combined with Mesenchymal Stem Cells or Neural Stem Cells for Chronic Spinal Cord Injury Repair | January 2016 | Phase I/II Enrolling by invitation | N/A | NCT02688049 China |
| Collagen scaffold + ESS (NR) | Safety and Efficacy of Functional Neural Regeneration Collagen Scaffold Transplantation Combined With Epidural Electrical Stimulation for Spinal Cord Injury Repair | August 2019 | Phase I/II Recruiting | N/A | NCT03966794 China |

**Abbreviations:** BCI (Brain Computer Interfacing); bMMCs (Bone Marrow Mononuclear Cells); MSCs (Mesenchymal Stem Cells); NP (Neuroprotective approach); NR (Neuroregenerative approach); NSCs (Neural Stem Cells); SCS (Spinal Cord Stimulation); SMF (Substitution of Motor Function approach); and SSMF (Substitution of Sensory and Motor Functions approach). N/A: not applicable/ not available.

**Table S2.** Major Findings Regarding GBMs Interfacing Neural Cells

| Nanomaterials | GBM fabrication method | Additional stimuli | Cell type/ model | Major Findings | REF |
|---|---|---|---|---|---|
| - MWCNTs (i.d. = 2 nm; o.d. = 13 nm)<br>- GO and rGO nanosheets (layer thickness = 1.2 nm; l.d. = from hundreds nm until several µm): | GO: Modified Hummers'method<br>rGO: reduction with L-ascorbic acid | Stromal cell-derived inducing activity method after 10 days | Mouse ESCs (*in vitro*) | - Only GO boosts dopamine neuron differentiation in a dose dependent manner<br>- 100 µg/mL is a non-toxic dosage while inducing the most accentuated effect on neuronal differentiation | 87 |
| GO nanosheets (layer thickness = 2.23 – 4.20 nm; l.d. = 615 – 814 nm) | Commercial | N/A | Mouse ESCs:<br>- *in vitro* (12 days)<br>- Teratoma formation *in vivo* after subcutaneous injection in a mouse model (4 weeks) | - Formation of embryonic bodies (8 days) and then cultured with GO (16 µg/mL, 4 days)<br>- GO nanosheets sustain self-renewal ability of stem cells by downregulating vinculin and, subsequently, depressing expression of mitogen-activated protein kinase<br>- Concentration-depend expression of pluripotency gene markers<br>- Cytocompatibility maintained below 32 µg/mL<br>- Preservation of cell morphology anddifferentiation potential *in vitro* and *in vivo* | 88 |
| - Multilayered GO nanosheets (thickness = 3.2 nm)<br>- Monolayered GO nanosheets (thickness = 0.7 – 1.2 nm= | Commercial | N/A | Mouse ESCs:<br>- *in vitro* – Teratoma formation *in vivo* after subcutaneous injection in a mouse model (4 weeks) | - Culture with GO nanosheets (16 µg/mL, 3 days) after formation of embryonic bodies<br>- Cytocompatibility maintained below 32 µg/mL<br>- GO nanosheets mostly located outside cell membranes<br>- Multilayered GO presents higher surface energy<br>- Superior adsorption of fibronectin by multilayered GO contributes to downregulation of integrin signaling, leading to cell self-renewal<br>- Multilayered GO maintains cell differentiation potential *in vitro* and *in vivo* | 89 |

| | | | | | |
|---|---|---|---|---|---|
| GO nanosheets (l.d. = 400 nm); | Modified Hummers' method | - GO functionalized with COOH groups<br>- Differentiation medium | Human fetal NSC neurospheres (*in vitro*) | - A concentration of 10 μg/mL induces 45 % of cell death<br>- Neurospheres connect to each other via neurite-like structures at 5 μg/mL and 10 μg/mL<br>- GO nanosheets outside of cell membranes, being rarely internalized<br>- GO nanosheets (5 μg/mL) maintain cell self-renewal or accelerate differentiation according to the applied culture conditions | 90 |
| GO nanosheets with several hydrodynamic sizes (l.d. x thickness):<br>- 417 nm x 22.5 nm<br>- 663 nm x 17.7 nm<br>- 1047 nm x 22.4 nm<br>- 4651 nm x 13.4 nm | Commercial | Differentiation medium | Mouse NSCs (*in vitro*) | - GO nanosheets (20 μg/mL) show high cytocompatibility independently of their size<br>- GO nanosheets with sizes of 663 nm x 17.7 nm and 4651 nm x 13.4 nm enhance cell self-renewal or neural differentiation, respectively<br>- Cells treated with GO of 4651 nm x 13.4 nm show an improved ability to migrate | 91 |
| - GO nanosheets (thickness = 1.2 nm; l.d. =2 μm)<br>- rGO nanosheets<br>- Single-layer GO nanoribbons (length = 10 μm; width = 50–200 nm)<br>- Single-layer rGO nanoribbons | - GO: modified Hummers' method<br>- GO nanoribbon: oxidative unzipping of MWCNTs<br>- rGO and nanoribbons: hydrazine reduction | N/A | Human MSCs (*in vitro*) | - Cytotoxicity is 30 % lower in GO nanosheets and nanoribbons comparatively to their reduced counterparts<br>- Cytotoxicity is time and concentration dependent<br>- rGO nanoribbons produce genotoxicity at 10 μg/mL after 1 h in culture, while rGO nanosheets exhibit similar effects at 100 μg/mL after 96 h<br>- Production of ROS is the prominent cytotoxic pathway triggered by nanosheets | 92 |
| - GO nanosheets (thickness = 0.7 nm; l.d. = 3.8 ± 0.4 μm)<br>- rGO nanosheets (thickness = 1.1 – 2.3 nm)<br>- rGO nanoplatelets (thickness = 1.1. nm; l.d. = 30 nm) | - GO: modified Hummers' method<br>- rGO: hydrazine reduction<br>- rGO nanoplatelets: sonication of covalently PEGylated GO + hydrazine reduction | PEGylation of GO before reduction for synthesizing nanoplatelets | Human MSCs (*in vitro*) | - Cytotoxicity is 30 % lower in GO nanosheets comparatively to their reduced counterparts<br>- Cytotoxicity is size, time and concentration dependent<br>- Nanoplatelets induce genotoxicity at low concentrations (0.1 and 1.0 μg/mL after 1 h)<br>- rGO shows negligible cytotoxicity even at 100 μg/mL after 1 h<br>- Production of ROS is the prominent cytotoxic pathway triggered by rGO | 93 |

| | | | | | |
|---|---|---|---|---|---|
| GO nanosheets with several sizes (mean diameter): 12.6, 40.8 and 327.6 nm | -Modified Hummers' method | N/A | Mouse ESCs (*in vitro*) | - GO with sizes below 20 nm suppresses cell growth and increases cell death<br>- All sizes affect cell proliferation in a concentration dependent manner<br>- GO diameter > 20 nm at 5 μg/ml as the optimal setup for cell-GO interactions | 94 |
| GQDs | ¿?¿?¿¿ | Differentiation medium | Human NSCs (*in vitro*) | - GQDs internalization via endocytosis in a concentration and time dependent manner<br>- Preservation of cell viability, proliferation, self-renewal capacity and differentiation potential<br>- Similar cell responses independently of concentration (1 - 100 μg/ml) | 98 |
| GQDs (diameter < 5 nm) | Modified graphite intercalation compounds method | N/A | - Human adipose-derived stem cells (*in vitro*)<br>- Subcutaneous transplantation of labelled cells in a rat model (*in vivo*) | - Preserved cell viability and metabolic activity *in vitro*<br>- *In vivo* cell tracking during 24 h post-implantation | 99 |
| GQDs (diameter = 3−5nm) | Commercial | Differentiation medium | Mouse ESCs (*in vitro*) | - GQDs are cytocompatible, inducing a cell viability higher than 80% even at large concentrations (*e.g.* 200 μg/mL)<br>- GQDs sustain self-renewal capability of ESCs<br>- GQDs (50 μg/mL) delay the differentiation into embryonic bodies by inhibiting DNA methylation of the pluripotency factor Sox2 | 102 |
| 2D nanosheets:<br>few-layer graphene<br>monolayer GO flakes | - Graphene: exfoliation of graphite + ball milling<br>- GO: oxidation of carbon fibres | N/A | Primary neurons isolated from embryonic brain cortices (*in vitro*) | - No significant differences between 1 μg/ mL and 10 μg/mL<br>- No deleterious impact on cell viability and morphology<br>- Nanomaterials internalized by cells follow endolysosomal pathways<br>- No substantial modification in neuronal activity after short- (24 h) and long-term (2 weeks) contact with graphene<br>- Long term exposure to GO inhibit neuronal activity, downregulate excitatory synapses and upregulate inhibitory synapses | 105 |

| | | | | | |
|---|---|---|---|---|---|
| GO nanosheets:<br>- large (l.d. = 10 - 15 µm)<br>- small (l.d. = 50 nm - 500 nm) | Modified Hummers' method + sonication | N/A | Hippocampal neurons and primary glial cells (*in vitro*) | - Larger GO flakes induce cytotoxicity for both neurons and glia<br>- Smaller GO flakes (10 µg/mL) influence neuronal activity by downregulating glutamate-mediated synapses<br>- Smaller GO flakes (10 µg/mL) increase the release of synaptic-like microvesicles from astrocytes | 106 |
| GO nanosheets (l.d. = 100 nm - 300 nm) | Modified Hummers' method | N/A | - Hippocampal neurons and organotypic hippocampal slices (*in vitro*)<br>- Intrahippocampal delivery using a rat model (*in vivo*) | - GO induces an initial increase of excitatory activity followed by an accentuated reduction<br>- Maintenance of cell viability *in vitro*<br>- After 72 h of injection, nanomaterials absent of the targeted area *in vivo* (50 µg/ mL)<br>- Tissue reactivity higher in the injection area and minimal in adjacent zones *in vivo* (50 µg/ mL) | 107 |
| - GO nanosheets (l.d. = 10-1800 nm)<br>- Functionalized CNTs<br>- Charged liposomes | Modified Hummers' method | N/A | Intracerebral injection in rats (*in vivo*) | After 7 days post-injection (0.5 µg/ µL and 1 µg/ µL):<br>- No significant differences between tested doses of carbon-based nanomaterials<br>- Absence of neurodegeneration or microglia activation<br>- GO nanosheets moderate inflammation | 108 |
| GO nanosheets (l.d. = 30–700 nm) | Modified Hummers' method + sonication | N/A | Intraspinal injection in zebrafish (*in vivo*) | After 2 days post-injection (0.9 mg/ ml):<br>- Maintenance of cell viability<br>- GO nanosheets augment inhibitory synapses rather than excitatory neurotransmissions, affecting locomotor activity | 109 |
| GO nanosheets (l.d. = 0.5 to several µm) | Commercial | N/A | GO translocation from the water to the brain of zebrafish larvae (*in vivo*) | After 24 h of exposure (0.01 µg/ L - 1 µg/ L):<br>- Parkinson's disease-like symptoms such as reduction of locomotive activity and loss of dopaminergic neurons | 110 |

| | | | | | |
|---|---|---|---|---|---|
| 2D nanosheets (size = 300 nm to few µm):<br>- GO<br>- rGO | - GO obtained from Grupo Antolin company (Spain)<br>- rGO : Thermal reduction | N/A | - Exposure of zebrafish embryos to GBMs (*in vivo*)<br>- Primary hippocampal cultures (*in vitro*) | *in vivo* (100 µg/mL):<br>- GO: early (after 2 h of exposure), but not persistent (absent after 24 h) inhibitory effects on the locomotor performance of zebrafish embryos<br>- rGO: delayed (after 4 h) and long-term (noticeable after 24 h) increase of the locomotor performance of zebrafish embryos induced by an augmentation of the synaptic activity<br>*in vitro* (10 µg/mL; 6-8 days in culture):<br>- rGO increases the neuronal network excitability | 111 |
| rGO nanosheets (size = 98.4 ± 21.3 nm and 189.9 ± 31.1 nm in water and culture medium, respectively) | Reduction with Vitamin C | N/A | PC12 cells line and embryonic primary cortical neurons (*in vitro*) | Up to 12 h of exposure (20 µg/mL):<br>- Intracellular oxidation of rGO via intracellular reactive oxygen species<br>- Depression of neurotransmission | 112 |
| rGO nanosheets (size = 342 ± 23.5 nm) | - Catalytic conversion + CVD<br>- Thermal reduction | N/A | Single i.v. dose (7 mg/ kg body weight) in rats (*in vivo*) | Up to 7 days post-injection:<br>- No evidence of significant CNS toxicity | 113 |
| rGO nanosheets (large = 472.08 ± 249.17 nm; small = 87.97 ± 30.83) | Modified Hummers' method | N/A | Administered to rats via oral gavage (60 mg/ kg) every 24 h for 5 consecutive days (*in vivo*) | First 3-4 days after the last administration:<br>- Short-term decrease in locomotor activity and neuromuscular coordination<br>- No significant long-term effects | 114 |
| GQDs (oval shape with thickness = 2 nm and diameter ~ 20 nm) | - Electrochemical exfoliation of graphite<br>- Thermal decomposition of citric acid | N/A | - i.p. injection in rats (10 mg/Kg/day) for 32 days (*in vivo*) | - GQDs increase MAPK/Akt signalling pathway and regulate the encephalitogenic Th1 immune response<br>- Reduced immune inflammation, axonal damage, demyelination and cell death in GQD-treated rats<br>- High survival rate of GQDs-treated neurons and oligodendrocytes when exposed to T-cell-mediated damage *in vitro* after 48 h | 115 |

| | | | | | |
|---|---|---|---|---|---|
| GQDs (thickness ~ 2 nm) | Carbon fibers under acidic and temperature conditions + filtration | N/A | - Primary cortical neurons (*in vitro*)<br>- i.p. injection in rats (50 µg/ animal) every 2 weeks for 6 months (*in vivo*) | GQDs induce disaggregation of Parkinson's α-synuclein mature fibrils<br>*in vivo*:<br>- Penetration across the blood-brain barrier without cytotoxicity<br>- Neuroprotection against α-synuclein<br>*in vitro*:<br>- Reduction of cell death, synaptic loss, Lewy body and Lewy neurite formation, and mitochondrial dysfunction<br>- Prevention of neuron-neuron transmission of α-synucleinopathy | 116 |
| 2D nanosheets:<br>- Graphene (l.d. = 500 nm to 2000 nm)<br>- GO (l.d. = 100 nm to 1500 nm) | - Graphene: exfoliation of graphite + ball milling<br>- GO: oxidation of carbon fibres | N/A | - Primary rat cortical astrocytes (*in vitro*)<br>- Co-culture with primary cortical neurons (*in vitro*) | - Maintenance of cell viability.<br>- Morphological cell variations from epithelioid to asymmetric shape with long processes<br>- GO internalization (10 µg/ml) substantially increase $K^+$ buffering and glutamate uptake<br>- GO-treated astrocytes increase maturation of co-cultured neurons and inhibitory synapses | 118 |
| 2D nanosheets (l.d. = 100 to 1500 nm):<br>- Few-layered graphene<br>- GO | - Graphene: exfoliation of graphite + ball milling<br>- GO: oxidation of carbon fibres | N/A | Primary rat cortical astrocytes (*in vitro*) | Up to 72 h of exposure (10 µg/ml):<br>- Preservation of viability, proliferation and reactivity<br>- Morphological cell transition from epithelioid to asymmetric shapes with long processes<br>- Graphene and GO generate distinct proteomic and lipidomic profiles<br>- GO augments cholesterol levels in cell membranes<br>- GO influences intracellular calcium dynamics | 119 |
| 2D nanosheets:<br>GO (l.d. = 50 nm to 500 nm | Modified Hummers' method | N/A | - Primary glial cultures from neonatal rat cortices (*in vitro*)<br>- Cortical neurons isolated from neonatal rat cortices (*in vitro*) | - Astrocytes exposed to GO (10 mg/ mL, 6 days) increase both production and release of microvesicles involved in intercellular communication<br>- Neurons exposed to GO-derived microvesicles (8-10 days) enhance synaptic activity (increase of postsynaptic currents frequency) together with a significant softening of the neuronal mechanical properties | 120 |
| 2D nanosheets:<br>- GO<br>- rGO | - GO: commercial<br>- rGO: Thermal reduction | N/A | RAW-264.7 macrophages (*in vitro*) | After 24 h of treatment with 1, 5, and 10 µg/mL:<br>- rGO triggers lower levels of reactive oxygen species and proinflammatory cytokines<br>- Only higher concentrations of GO induce a significant expression of pro-inflammatory CD80 marker | 121 |

| | | | | | |
|---|---|---|---|---|---|
| 2D nanosheets:<br>GO (l.d. = 30–700 nm) | Modified Hummers' method + sonication | N/A | 3D mouse organotypic spinal cord cultures (*ex vivo*) | - Long-term GO exposure (14 days) does not compromise neuronal viability.<br>- High concentrations of GO (25 and 50 µg/ mL) downregulate both excitatory and inhibitory synapses<br>- Maintenance of astrocyte density<br>- Increase of microglia proliferation without significant release of pro-inflammatory molecules | 122 |

**Abbreviations:** Abbreviations: CVD (Chemical Vapor Deposition); ESCs (Embryonic Stem Cells); GO (Graphene Oxide); GQDs (Graphene Quantum Dots); hNSCs (Human Neural Stem Cells); l.d. (lateral dimensions); MSCs (Mesenchymal Stem Cells); MWCNTs (Multi Walled Carbon Nanotubes); NSCs (Neural Stem Cells) and rGO (Reduced Graphene Oxide). N/A: not applicable/ not available.

**Table S3.** Most Remarkable Strategies Regarding the use of 3D Graphene-based Scaffolds Targeting SCI

| 3D Architecture | Additional functionalization/ stimuli | Cell type/ model | Major Findings | REF |
|---|---|---|---|---|
| Porous system:<br>Polydimethylsiloxane + graphene | N/A | Hippocampal cells culture (*in vitro*) | - Pore size: 100–200 µm<br>- Highly interconnected neural networks with neurons and glia<br>- Functional synaptic activity, including synchronization of active neurons<br>- Modification of GABAergic inhibition maturation | 279 |
| Porous system:<br>Graphene | Poly-L-ornithine + Matrigel coating | Hippocampal neurons (*in vitro*) | - Pore size: 100 - 500 µm.<br>- Highly interconnected neural networks with neurons and glia<br>- Synchronous neuronal activity, including highly synchronized and moderately synchronized | 280 |
| Porous system:<br>Graphene | Electrical stimulation | Neural progenitor cells (*in vitro*) | - Accurate 3D geometrical rearrangement of graphene building blocks<br>- Smaller graphene skeleton widths (10 µm and 50 µm of spacing) boost neuronal differentiation and favour astrocytic processes<br>- Electrical stimulation modulates intracellular $Ca^{2+}$ concentration | 281 |
| Porous system:<br>Graphene | N/A | Cortical neurons from rats (*in vitro*) | - Pore size = 20 µm; Graphene building blocks with a skeleton width = 20 µm and orientation angles of 90°<br>- Elongated axons and dendrites<br>- Ramified astrocyte morphology<br>- Functional neuronal network over long distances after 8 days of cell culture. | 282 |

| | | | | |
|---|---|---|---|---|
| Porous system:<br>rGO | - Hexamethylene diisocyanate vapours<br>- Thermal reduction (200 °C, 30 min) | C6 hemisection in rats (*in vivo*) | - Suitable integration into host tissue<br>- Collagen infiltration<br>- Cell migration into scaffold, particularly non neuronal cells<br>- M2-like macrophages present into both interfaces (predominantly) and scaffold interior<br>- No signs of systemic toxicity in major organs after 10 days post-implantation | 283 |
| Porous system:<br>rGO | - Hexamethylene diisocyanate vapours<br>- Thermal reduction (200 °C, 30 min) | ENPCs (*in vitro*) | - Pore size = 154 ± 34 x 43 ± 11 μm<br>- Differentiation of ENPCs into neurons and glia<br>- Highly viable, interconnected and functional neural networks | 284 |
| Porous system:<br>rGO | Thermal reduction (200 °C, 30 min) | C6 hemisection in rats (*in vivo*) | - SCI stabilization and sealing<br>- Suitable integration into host tissue<br>- Certain amount of axonal outgrowth inside scaffold<br>- Macrophages and fibroblasts prominent within scaffold<br>- Enhanced angiogenesis at both periphery and inside scaffold after 30 days post-implantation | 285 |
| Porous system:<br>rGO | Thermal reduction (200 °C, 30 min) | C6 hemisection in rats (*in vivo*) | - Pore size: 31.88 ± 19.43μm<br>- Preservation of rat spontaneous behaviour<br>- Suitable integration into host tissue<br>- Structural stabilization of the lesion<br>- Collagen infiltration<br>- Outgrowth of myelinated excitatory axons inside scaffold<br>- Angiogenesis<br>- Insights of scaffold biodegradation (intracellular presence of rGO sheets) without toxic effects in major organs after 120 days post-implantation | 271 |
| Porous system:<br>Collagen + amino-functionalized graphene cryogel | Electrical stimulation | - Bone marrow derived MSCs (*in vitro*)<br>- Raw 264.7cells (*in vitro*) | - Pore size: 60 - 200 μm<br>- Enhanced MSCs adhesion, proliferation and neuronal differentiation<br>- Preferential M2 polarization of macrophages<br>- Electrical stimulation enhanced repair and immune-modulatory properties of MSCs and increased expression of neuronal markers (MAP-2 and β-tubulin III) | 289 |

| | | | | |
|---|---|---|---|---|
| | | - 3D organotypic spinal slices culture from rats (*ex vivo*) | - Cell proliferation from 3D organotypic spinal explants, spreading across scaffold pores | |
| Porous system:<br>Chitosan + GO | Genipin crosslinking | - PC12 cells (*in vitro*)<br>- T9 complete transection in rats (*in vivo*) | - No difference in PC12 viability relatively to chitosan after 3 days<br>- Pore size variation from ~ 37 µm to ~ 79 µm after 10 weeks post-implantation<br>- *in vivo* angiogenesis and tissue growth across the pores and enhanced locomotor performance as measured by BBB (score = 8 – 9) | 290 |
| Fibrous-Porous system:<br>Bacterial cellulose + graphene | N/A | - NSCs (*in vitro*)<br>- Primary cortical neurons (*in vitro*) | - Enhanced adhesion, growth and proliferation of NSCs<br>- Maintenance of stemness degree of NSCs<br>- Neuronal differentiation of NSCs generates interconnected and functional networks<br>- Bacterial cellulose nanofibres supports denser, functional and viable neuronal networks | 291 |
| Fibrous-Porous system:<br>PCL-gelatin + rGO | PLL coating | ENPCs (*in vitro*) | - Pore size customized according to nanofibres chemical composition<br>- Enhanced cell adhesion<br>- Differentiation into neuronal and non-neuronal cells<br>- Generation of highly viable and interconnected neural networks<br>- Neurite elongation and outgrowth directed by electrospun PCL-nanofibres located onto rGO surface | 292 |
| rGO microfibres + gelatin hydrogel to facilitate *in vivo* implantation | - Hydrothermal reduction (220 °C, 2 h)<br>- Coating with either PLL or N-cadherin (*in vitro*) | - ENPCs and meningeal fibroblasts (*in vitro*)<br>- C6 hemisection in rats (*in vivo*) | - Microfibres diameter customized according to hydrothermal parameters<br>*in vitro*:<br>- Enhanced ENPCs differentiation in both neurons and glia<br>- Generation of interconnected and functional neural networks<br>- N-cadherin coating prevents extensive fibroblasts adhesion<br>*in vivo*:<br>- Suitable integration into host tissue<br>- Enhanced neuroprotective responses relatively to injury alone<br>- Neuronal and non-neuronal (preferentially) cell colonization of the scaffold<br>- No sighs of toxicity after 10 days post-implantation | 293 |

| | | | | |
|---|---|---|---|---|
| rGO microfibres | - Hydrothermal reduction (220 °C, 2 h)<br>- PLL coating for ENPCs. | - RAW-264.7 macrophages (*in vitro*)<br>- ENPCs (*in vitro*) | - Microfibres diameter = 106 ± 3 μm<br>- Decrease of RAW-264.7 proliferation without affecting viability<br>- Enhanced intracellular ROS content<br>- Preferential polarization towards M1 and M2 phenotypes after 24 h and 48 h, respectively<br>- Initial decrease of TNF-α and IL-6 (24 h), followed by increase of IL-6 after 48 h<br>- M1 and M2 macrophages presented rounded and elongated phenotypes, respectively<br>- Successful differentiation of ENPCs into both neurons and glia, generating highly interconnected neural networks | 294 |
| rGO microfibers + gelatin hydrogel to facilitate *in vivo* implantation | - Hydrothermal reduction (220 °C, 2 h)<br>- PLL coating (*in vitro*) | - ENPCs (*in vitro*)<br>- C6 hemisection in rats (*in vivo*) | *in vitro*:<br>- Enhanced ENPCs adhesion and differentiation into both neuronal and non-neuronal cells<br>- Generation of interconnected neural networks<br>*in vivo:*<br>- Suitable integration into host tissue<br>- Infiltration of connective tissue<br>- Angiogenesis *in vivo*<br>- No significant augmentation of inflammatory responses<br>- Neurons identified close by microfibers<br>- Similar functional behavioural responses relatively to control after 120 days post-implantation | 295 |
| Fibrous system:<br>Poly(l-lactic acid-co-caprolactone) printed microfibrous scaffold | - GO coating + *in situ* reduction (ascorbic acid at 70 °C, 4 h)<br>- Electrical stimulation | - PC-12 (*in vitro*)<br>- Primary hippocampal neurons (*in vitro*) | - Smaller diameter microfibres (17 μm) enhance neurite outgrowth (direction and length). Other diameters (72 or 150 μm) enable more accentuated neurites branching and sprouting<br>- Electrical stimulation enhances directional neurites growth | 302 |
| Electrospun fibrous system:<br>Silk fibroin + graphene | N/A | Spinal cord neurons (*in vitro*) | - Viable neuronal cultures<br>- Enhanced neurite outgrowth<br>- High expression level of Netrin-1 | 307 |

| | | | | |
|---|---|---|---|---|
| Electrospun fibrous system:<br>PLGA + GO | Delivery of insulin-like growth factor 1 (IGF-1) and brain-derived neurotrophic factor (BDNF) | - NSCs (*in vitro*)<br>- T9 hemisection in rats (*in vivo*) | - Enhanced adhesion, proliferation and survival of NCSs<br>- NSCs differentiated into neurons or astrocytes when cultured onto PLGA-GO-IGF-BDNF or PLGA-GO-IGF, respectively<br>- Reduction of cavity area<br>- Functional recovery after 4 weeks post-implantation as measured by BBB (score ~ 17) | 308 |
| Injectable hydrogel:<br>GO + diacerein-terminated four-armed PEG | N/A | - BV-2 microglial cells (*in vitro*)<br>- Primary mouse astrocytes (*in vitro*)<br>- T9 moderate crushing injury in rats (*in vivo*) | *in vitro*:<br>- Inhibition of inflammatory responses and astrocyte hyperactivation<br>*in vivo*:<br>- Suitable integration into host tissue<br>- Reduction of injured area and inflammation<br>- Neuronal and axonal regeneration<br>- Functional recovery after 28 days post-injection as measured by BBB (score = 13 ± 3). | 310 |
| Gelatin methacrylate hydrogel containing functionalized poly (lactic-co-glycolic acid) microcapsules | - Layer-by-layer assembly with rGO and silk-PLL<br>- Delivery of Neurotrophin-3 | - NSCs (*in vitro*)<br>- T8 hemisection in rats (*in vivo*) | - Enhanced migration and neuronal differentiation of endogenous NSCs<br>- Mitigated inflammation<br>- Functional recovery after 35 days post-implantation as measured by BBB (score = 16 ± 2) | 311 |

**Abbreviations:** BBB (Basso, Beattie, Bresnahan locomotor scale); ENPCs (Embryonic Neural Progenitor Cells); GO (Graphene Oxide); NSCs (Neural Stem Cells); PEG (Polyethylene glycol); PLGA (Poly(lactic-co-glycolic acid); PLL (Poly-L-lysine) and rGO (Reduced Graphene Oxide). N/A: not applicable/not available.
